# Remediation of Metal Oxide Nanotoxicity with a Functional Amyloid

**DOI:** 10.1002/advs.202310314

**Published:** 2024-04-06

**Authors:** Yue Wang, Xiufang Liang, Nicholas Andrikopoulos, Huayuan Tang, Fei He, Xiang Yin, Yuhuan Li, Feng Ding, Guotao Peng, Monika Mortimer, Pu Chun Ke

**Affiliations:** ^1^ School of Biomedical Sciences and Engineering Guangzhou International Campus South China University of Technology Guangzhou 510006 China; ^2^ Nanomedicine Center Great Bay Area National Institute for Nanotechnology Innovation 136 Kaiyuan Avenue Guangzhou 510700 China; ^3^ Drug Delivery Disposition and Dynamics Monash Institute of Pharmaceutical Sciences Monash University 381 Royal Parade Parkville VIC 3052 Australia; ^4^ Department of Engineering Mechanics Hohai University Nanjing 211100 China; ^5^ Department of Physics and Astronomy Clemson University Clemson SC 29634 USA; ^6^ College of Environmental Science and Engineering Key Laboratory of Yangtze River Water Environment Tongji University 1239 Siping Road Shanghai 200092 China; ^7^ Liver Cancer Institute Zhongshan Hospital Key Laboratory of Carcinogenesis and Cancer Invasion Ministry of Education Fudan University Shanghai 200032 China; ^8^ Laboratory of Environmental Toxicology National Institute of Chemical Physics and Biophysics Akadeemia tee 23 Tallinn 12618 Estonia

**Keywords:** functional amyloid, ion release, metal oxide nanoparticle, sequestration, toxicity

## Abstract

Understanding the environmental health and safety of nanomaterials (NanoEHS) is essential for the sustained development of nanotechnology. Although extensive research over the past two decades has elucidated the phenomena, mechanisms, and implications of nanomaterials in cellular and organismal models, the active remediation of the adverse biological and environmental effects of nanomaterials remains largely unexplored. Inspired by recent developments in functional amyloids for biomedical and environmental engineering, this work shows their new utility as metallothionein mimics in the strategically important area of NanoEHS. Specifically, metal ions released from CuO and ZnO nanoparticles are sequestered through cysteine coordination and electrostatic interactions with beta‐lactoglobulin (bLg) amyloid, as revealed by inductively coupled plasma mass spectrometry and molecular dynamics simulations. The toxicity of the metal oxide nanoparticles is subsequently mitigated by functional amyloids, as validated by cell viability and apoptosis assays in vitro and murine survival and biomarker assays in vivo. As bLg amyloid fibrils can be readily produced from whey in large quantities at a low cost, the study offers a crucial strategy for remediating the biological and environmental footprints of transition metal oxide nanomaterials.

## Graphical abstract

This study presents a facile scheme of detoxifying transition metal oxide nanomaterials by a functional beta‐lactoglobulin (bLg) amyloid. The biocompatible whey protein amyloid acted as a metallothionein mimic to sequester toxic metal ions dissolved by CuO and ZnO nanoparticles, mediated by chelation as well as electrostatic interaction between the metal oxide nanoparticles and the amyloid fibrils.

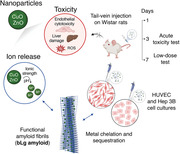

## 1 Introduction

The application of engineered nanomaterials in research and industry has been rapidly growing, which will result in their eventual discharge and exacerbate their environmental burden.^[^ ^1^, ^2^, ^3^ ^]^ Accordingly, understanding the environmental health and safety of nanomaterials (NanoEHS) has evolved into a multidisciplinary research frontier concerning the fate and implications of nanomaterials in living systems in the ecosphere, harnessing the principles and methodologies of toxicology, materials, and environmental science and technology.^[^ ^4^, ^5^ ^]^ Despite considerable research over the past two decades, NanoEHS remains a field known for identifying problems but offering few solutions.^[^ ^6^ ^]^

Transition‐metal oxides are widely employed in pharmaceuticals, construction, electronics, and cosmetics because of their appealing physical and physicochemical properties.^[^ ^7^, ^8^, ^9^ ^]^ Their inevitable environmental discharge poses a realistic risk to animals and humans. CuO and ZnO nanoparticles, two of the most representative nanomaterials, have been extensively studied over the past 15 years. Inflammation, apoptosis, and necrosis in the brain, liver, kidneys, spleen, gut, and vasculature of animal models have been considered as their major toxicity indicators.^[^ ^10^, ^11^, ^12^ ^]^ The toxicity mechanisms elicited by CuO and ZnO nanoparticles range from genotoxicity^[^ ^13^ ^]^ and oxidative stress to cell membrane damage and mitochondrial injury,^[^ ^14^, ^15^ ^]^ triggered by ion release from the nanoparticle hosts as a common mode of action.^[^ ^16^, ^17^ ^]^ Indeed, several studies have indicated ion release as the primary route of CuO and ZnO nanotoxicity.^[^ ^18^, ^19^ ^]^

Amyloid proteins play a causative role in the pathogenesis of neurodegenerative and metabolic diseases.^[^ ^20^, ^21^ ^]^ By contrast, functional amyloids derived from chaperone proteins and bacterial extracellular matrices possess unique physicochemical properties, and can act as building blocks for the construction of novel functional materials and nanocomposites.^[^ ^22^, ^23^, ^24^, ^25^ ^]^ In terms of biocompatibility, recent studies have demonstrated that amyloid‐based gels induced minimal cytotoxicity, and could serve as scaffolds for cell culture and growth,^[^ ^26^, ^27^ ^]^ whereas digested amyloid fibrils derived from beta‐lactoglobulin (bLg),^[^ ^28^ ^]^ a whey protein, elicited no toxicity in Caco‐2 cell lines, *Caenorhabditis elegans*, and Kunming mice.^[^ ^29^ ^]^ Furthermore, amyloid seeds derived from food proteins (including bLg) did not exacerbate Aβ_1‐42_ aggregation, unlike pathogenic proteins which tend to initiate amyloidogenesis through cross‐seeding.^[^ ^30^ ^]^ In terms of biomedical applications, bLg amyloid fibrils have recently been employed for nutritional iron fortification in male Wistar rats^[^ ^31^ ^]^ and, when complexed with hyaluronic acid, for forming coacervates and being administered orally for gastric ulcer protection in male *Rattus norvegicus*.^[^ ^32^ ^]^ In addition, monomeric bLg protein inhibits the primary nucleation, elongation, and toxicity of amyloid beta in connection with the pathology of Alzheimer's disease,^[^ ^33^ ^]^ whereas bLg‐amyloid‐fragment‐coated gold nanoparticles effectively block the amyloidogenesis pathway of human islet amyloid polypeptides associated with type 2 diabetes via the formation of functional‐pathogenic double‐protein coronae.^[^ ^34^ ^]^ In environmental engineering, bLg amyloid fibrils have shown remarkable efficiency against heavy metal pollutants in sewage water purification.^[^ ^35^ ^]^

**Scheme 1:**
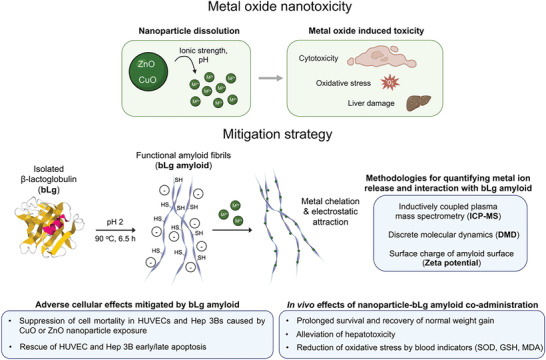
Ion release and in vitro and in vivo toxicity of metal oxide nanomaterials mitigated by the bLg functional amyloid. Cu^2+^ and Zn^2+^ ions released by CuO and ZnO nanomaterials, respectively, can be sequestered by the functional bLg amyloid, as quantified by ICP‐MS. Mechanistic insights into bLg‐amyloid–metal binding were offered by DMD simulations and a zeta potential analysis. The protective potential of the bLg amyloid against cell death was assessed in endothelial HUVEC and Hep 3B cultures and in vivo with a rat model exposed to the metal oxide nanomaterials.

In this study, we demonstrated a new usage of functional amyloids for biological and environmental remediation, where bLg amyloid fibrils sequestered metal ions to suppress the toxicity of CuO and ZnO nanoparticles, two major types of metal oxide nanomaterials with significant biological and environmental footprints (**Scheme** **1**). High‐resolution structural studies have revealed that amyloid fibrils formed by different amyloid proteins comprise in‐register, parallel β‐sheets with strands aligned perpendicular to the fibril axis. These β‐sheets pack against each other to form a hydrophobic cross‐core and expose their polar residues.^[^ ^36^ ^]^ Hence, with the same residues of different chains strung together, ion‐binding residues, such as histidine, acidic amino acids, cysteine, and methionine,^[^ ^37^ ^]^ in an amyloid fibril offer ample sites along the fibril to chelate divalent and multivalent metal ions. In this study, we used inductively coupled plasma mass spectrometry (ICP‐MS) to characterize the dose‐ and time‐dependent release of Cu^2+^ and Zn^2+^ from host nanoparticles, and their collection by bLg amyloid fibrils. Discrete molecular dynamics (DMD) simulations and zeta potential analyses were performed to gain insights into the underlying mechanisms of metal‐ion binding. Human umbilical vein endothelial cells (HUVECs) and human hepatocellular carcinoma (Hep 3B) cells were cultured to assess the protective potential of bLg amyloid fibrils against the uptake and cell death elicited by CuO and ZnO nanoparticles. bLg amyloid fibrils suppressed toxicity and restored the survival of rats exposed to metal oxide nanomaterials in vivo (Scheme 1). This study offers a viable strategy for the biological and environmental remediation of the toxic burden of transition metal oxide nanomaterials.

## 2 Results and Discussion

### 2.1 Characterizations of CuO and ZnO Nanoparticles and bLg Amyloid

**Figure 1:**
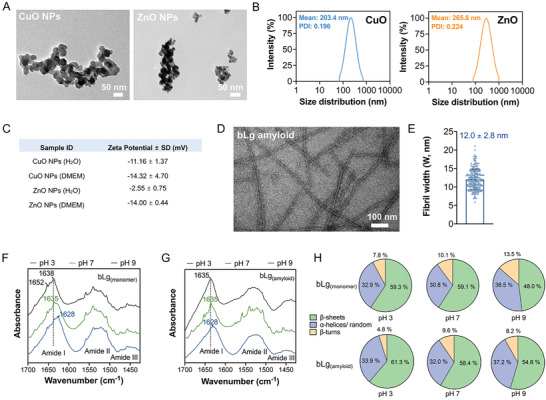
Material characterizations. A) TEM images of CuO and ZnO nanoparticles (NPs). Scale bars: 50 nm. B) Size distribution (d, nm) of CuO and ZnO nanoparticles (1.25 mm; Note that the molar concentrations of metal oxide nanoparticles hereafter refer to the concentrations of the metal oxide molecules constituting the nanoparticles, for easy comparisons with metal salts and ions) in Milli‐Q water determined by intensity (%) (n = 1). C) ζ‐potential values (mean ± SD) of CuO and ZnO nanoparticles (1.25 mm) in Milli‐Q water and DMEM medium (n = 3). D) Morphology of bLg amyloid fibrils at a neutral pH as indicated by TEM. Scale bar: 100 nm. E) Fibril width (W, nm) analysis conducted on a large set of individual fibrils (n = 200) through ImageJ. F–H) Secondary structure analysis (%) (β‐sheet, α‐helical/random, β‐turn content) of bLg monomeric (F) and amyloid species (G) at pH 3, 7, and 9 derived from the ATR‐FTIR spectra (1700–1426 cm^−1^) presented in panels F and G. Secondary structure analysis (%) (H) was acquired upon the deconvolution of the amide I band presented in Figure S2, Supporting Information.

CuO and ZnO nanoparticles have been extensively studied because of their environmental prevalence and associated cytotoxicity arising from dissolution and ion release in aqueous environments.^[^ ^38^ ^]^ Both types of nanoparticles exhibited irregular spherical shapes ranging between 30 and 50 nm (**Figure** **1**A) and displayed some degree of agglomeration, as indicated by dynamic light scattering (DLS) size distributions with low polydispersity indices (< 0.23) (Figure 1B). The nanoparticles were negatively charged in Milli‐Q water and Dulbecco's modified eagle medium (DMEM), as indicated by their negative ζ‐potential values (Figure 1C). This anionic nature mainly arose from the greater electronegativity (χ) of oxygen (χ = 3.5) than that of copper and zinc (χ < 2).

bLg as a functional protein undergoes partial denaturation and aggregation at high temperatures (>70 °C) driven by hydrophobic interactions. Low ionic strength and an acidic pH (2–3), that is, a pH lower than the isoelectric point (pI) of the protein (pI = 5.3), play crucial roles in the formation of bLg amyloid fibrils, with fast aggregation kinetics and high conversion rates at high temperatures.^[^ ^39^ ^]^ Specifically, electrostatic repulsion triggered by low pH and acidic hydrolysis promotes interactions between N‐terminal peptide regions with an amyloidogenic propensity^[^ ^40^, ^41^, ^42^ ^]^ to favor highly ordered aggregation. Herein, the preparation of bLg amyloid fibrils was based on previous reports,^[^ ^31^, ^43^, ^44^ ^]^ and mature bLg amyloid fibrils were obtained upon incubation at 90 °C for 6.5 h. Size‐exclusion chromatography‐diode array detection (SEC‐DAD) analysis of the collected supernatants (bLg monomers) upon centrifugation revealed a high yield of amyloid conversion (94.9%) (Figure S1, Supporting Information). Transmission electron microscopy (TEM) (Figure 1D) showed that, upon purification, the bLg fibrils exhibited a typical amyloid‐like morphology with an average width of 12.0 ± 2.8 nm (n = 200) (Figure 1E). Attenuated total reflection–Fourier‐transform infrared spectroscopy (ATR‐FTIR) was employed to study the secondary structures of the bLg monomer and amyloid species within a range of pH values (3, 7, and 9) after ATR‐FTIR peak deconvolution (Figure 1F–H and Figure S2, Supporting Information). The amide I region (1700–1600 cm^−1^) was assigned for this purpose based on its high intensity and sensitivity to variations in protein conformation (Figure S2, Supporting Information).^[^ ^45^ ^]^ The amide I absorbance band is mainly associated with the C═O stretching vibrations of the amide bonds. The assigned wavenumber regions of proteins based on their conformation are as follows: α‐helix (1648–1657 cm^−1^), β‐sheet (1623–1641 cm^−1^, 1674–1695 cm^−1^), random (1642–1657 cm^−1^), and β‐turn (1662–1686 cm^−1^).^[^ ^45^ ^]^ Purified bLg monomers and amyloid samples were readily dissolved in Milli‐Q water and exhibited an acidic pH of 3 upon HCl pretreatment. In its native state, bLg assumes a prevailing β‐sheet conformation, which can be attributed to its eight‐stranded antiparallel β‐barrel among one α‐helix (Scheme 1).^[^ ^46^ ^]^ The amide I band maximum peak (1628 cm^−1^) of bLg monomers at pH 3 further confirmed their high β‐sheet content (59.3%) (Figure 1F,H). In comparison, bLg amyloid species at an acidic pH exhibited a slightly elevated β‐sheet content of 61.3% with a similar peak maximum (1628 cm^−1^) (Figure 1G,H). The conformations of both protein species were monitored at physiological conditions upon incubation at 37 °C. Secondary structure analysis of the bLg monomers did not reveal any significant conformational transitions, suggesting that they were relatively stable in both neutral and acidic environments (Figure 1H and Figure S2, Supporting Information). In contrast, the β‐sheet content of the bLg amyloid at a neutral pH experienced a small reduction of 2.9% compared with that at an acidic pH (Figure 1H and Figure S2, Supporting Information), whereas TEM imaging revealed no significant morphological alterations for the fibrils (Figure 1D). The conformational stabilities of both the monomeric and fibrillar bLg species were further assessed under denaturing conditions in an alkaline environment. Specially, pH 9 and subsequent incubation at 70 °C induced a partial denaturation of bLg monomers, resulting in the unfolding of the helical structure, which underwent a transition to a more disordered conformation. This effect was indicated by the elevated absorbance peaks at 1646 and 1652 cm^−1^ and the band shift to a higher wavenumber of 1638 cm^−1^ in the spectra (Figure 1G). Such a conformational transition elevated the α‐helical/random content by 7.7%, followed by a 11.1% reduction in the β‐sheet content compared with that of the monomer sample at a neutral pH (Figure 1H). For the amyloid fibrils, the absorbance peaks in the assigned α‐helical/random wavenumber region did not exhibit any notable increases. The 3.8% decrease in the β‐sheet content (Figure 1H) could be due to the thermal degradation and shortening of bLg amyloid fibrils at high temperatures.^[^ ^47^ ^]^

### 2.2 Copper and Zinc Ion Sequestration by bLg Amyloid

**Figure 2:**
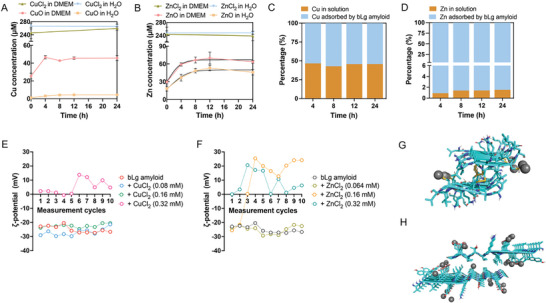
Metal ion adsorption capacity of bLg amyloid. (A‐D) Evaluation of metal ion sequestration by the bLg amyloid through ICP‐MS. A,B) Elemental (Cu, Zn) quantifications of collected supernatants from either nanoparticle (CuO, ZnO) suspensions or M (II) chloride salt (CuCl_2_, ZnCl_2_) solutions upon incubation for 0–24 h and centrifugation (20 000 *g*/15 min). Nanoparticles and metal chloride salts were dispersed or dissolved at 0.25 mm in Milli‐Q water or DMEM medium. The black lines in panel B indicate logarithmic trend lines. C,D) Elemental (Cu, Zn) adsorption ratio (%) of metal species in the presence of the bLg amyloid (5 mg mL^−1^) in DMEM medium upon incubation for 4–24 h and centrifugation (20 000 *g*/40 min). Nanoparticle dispersions at 0.25 mm in DMEM medium (without the bLg amyloid) at each respective timepoint were used as a reference for estimating the dissolved Cu and Zn contents in the supernatants (data in panels A and B). E,F) ζ‐potential (mV) values for the bLg amyloid (5 mg mL^−1^) samples in the presence and absence of CuCl_2_ and ZnCl_2_ collected over a 10‐measurement course (three cycles per measurement). Anionic bLg amyloid (5 mg mL^−1^) was suspended in metal salt solutions of various concentrations (CuCl_2_, 0.08–0.32 mm; ZnCl_2_, 0.064–0.32 mm) and allowed to incubate for 10 min. Upon centrifugation (20 000 *g* over 15 min) and pellet collection‐redispersion, the electrostatic potential at the slipping plane of the bLg amyloid surface was acquired through ζ‐potential (mV) measurements. G) The amyloid‐forming segment ^117^LACQCL^122^ and H) the negatively charged segment ^41^VYVEELKPTPEG^52^ were selected to model bLg amyloid fibrils. The fibrils were plotted as cartoons with the side chains shown in sticks and colored by elements. The Zn^2+^ ions are shown in black spheres.

Several studies have indicated that dissolution, in connection with distinct physicochemical properties, such as size, shape, charge, and surface composition, plays a crucial role in the toxicity of metal oxide nanomaterials.^[^ ^48^, ^49^, ^50^ ^]^ High dissolution rates of nanoparticles in cell culture media can be influenced by the high ionic strength and anions controlling the shift of the ion–particle equilibrium.^[^ ^51^ ^]^ Herein, the dissolution of CuO and ZnO nanoparticles (0.25 mm) into divalent metal ions in DMEM and Milli‐Q water was investigated for their supernatants using ICP‐MS after 0, 4, 8, 12, and 24 h of incubation at a neutral pH (**Figure** **2**A,B). For the error evaluation and exclusion of significant metal contributions to our measurements by solvents, equally concentrated Cu^2+^ and Zn^2+^ salt solutions (0.25 mm) were analyzed under identical conditions as the nanoparticle supernatants (Figure 2A,B). Both CuO and ZnO nanoparticles exhibited greater solubility in DMEM than in Milli‐Q water because of the higher content of chloride, phosphate, bicarbonate, and sulfate anions in DMEM (Figure 2A,B). Specifically, incubation for 4 h in DMEM and subsequent centrifugation resulted in a 14.5‐fold increase in the solubilized metal ions for CuO nanoparticles and a 1.6‐fold increase for ZnO nanoparticles in DMEM compared with those in H_2_O (Figure 2A,B). Beyond the incubation duration of 4 h, no significant variation in the aqueous Cu content was observed (Figure 2A,B). In contrast, the dissolution of ZnO nanoparticles in DMEM followed a logarithmic trend over 0–24 h, with a greater dissolution rate constant in DMEM (*k =* 0.58) than in water (*k =* 0.44) (Figure 2A,B).

On the other hand, bLg amyloid fibrils possess excellent metal‐binding properties and the capacity to remove heavy metals, such as lead, palladium, platinum, silver, and nickel.^[^ ^28^, ^35^, ^52^, ^53^ ^]^ Compared with bLg monomers, the greater metal sequestration efficiency of bLg amyloid structures has been attributed to their larger surface‐to‐volume ratio through the occurrence of different intermolecular interactions (ligand binding function, electrostatics). The high stability and resistance of bLg amyloid fibrils to biological clearance under highly acidic conditions further enable their environmental and biomedical applications.^[^ ^31^, ^35^, ^54^ ^]^ Similarly, the results in our study also confirmed that the bLg amyloid exhibited a high affinity for copper and zinc in Milli‐Q water, removing 82.2% and 99.5% of copper and zinc ions over incubation for 24 h, respectively (Figure S3, Supporting Information). To investigate the adsorption capabilities of the bLg amyloid toward Cu^2+^ and Zn^2+^ in cell culture media, CuO and ZnO nanoparticles (0.25 mm) with or without the bLg amyloid were incubated in DMEM for 4, 8, 12, and 24 h (Figure 2C,D). Following ultracentrifugation, ICP‐MS analysis was conducted to measure the Cu^2+^ and Zn^2+^ content in the supernatants. The bLg amyloid efficiently adsorbed Cu^2+^ and Zn^2+^ released by the CuO and ZnO nanoparticles, respectively (Figure 2C,D). Specifically, the bLg amyloid removed ≈55% of Cu^2+^ at 4 h and 98% of Zn^2+^ between 4 and 24 h. The ligand‐binding function of the bLg amyloid may depend on its single available Cys‐121 residue to chelate divalent and trivalent metal species.^[^ ^55^ ^]^ In addition, there are 36 negatively charged residues (26 Glu and 10 Asp) per bLg protein (pI: 5.3) that could contribute to the electrostatic attraction of metal ions at a neutral pH. According to the Pearson's hard‐acid soft‐base theory,^[^ ^56^ ^]^ soft acid metal ions with a lower ionic charge and greater ionic size tend to bind through thiol–metal coordination because of the larger size and lower charge density of sulfur atoms compared with those of oxygen and nitrogen. In contrast, small and highly charged hard metal ions preferentially interact with bLg via electrostatic interactions. In this study, Cu^2+^ and Zn^2+^ exhibit borderline properties, enabling them to adopt both hard and soft characteristics. Atomic structural studies have revealed that the inter‐protofilament distance of functional and pathogenic amyloid fibrils is on the order of 4.6 to 4.8 Å.^[^ ^20^ ^]^ This refers to ≈8.6 × 10^4^ monomers per two intertwined protofilaments for each amyloid fibril based on the bLg fibrillar contour lengths acquired through atomic force microscopy.^[^ ^44^ ^]^ In this context, it was estimated, based on the ICP‐MS analysis results (Figure 2C,D), that such bLg monomer populations could host ≈8 × 10^3^ Cu and 2 × 10^4^ Zn ions after 24 h of incubation.

To further examine whether electrostatic interactions could act synergistically with bLg metal chelation for multimodal metal sequestration, we incubated the bLg amyloid in Cu and Zn salt solutions at a range of concentrations: CuCl_2_ (0.08–0.32 mm) and ZnCl_2_ (0.064–0.32 mm) (Figure 2E,F). Upon incubation for 10 min and centrifugation (20 000 *g* for 15 min), the collected pellets of the bLg amyloid were redispersed in Milli‐Q water, and the corresponding electrostatic potentials at the slipping plane of the amyloid interface were measured through 10 consecutive ζ‐potential measurements. bLg amyloid fibrils possess a pI value of ≈5.3, and hence, they displayed a negative surface charge between −20 and −25 mV during the measurements at a neutral pH (Figure 2E,F). The selected metal salt concentrations were based on previously acquired ICP‐MS results, which indicated that the bLg amyloid has a higher binding affinity for Zn^2+^ than for Cu^2+^. Sample groups were also divided based on the nonsaturated (< 0.27 mm) and saturated (> 0.27 mm) presence of metals compared with the bLg amyloid. For CuCl_2_ incubated samples, no apparent change in the overall net charge of the bLg amyloid (< −20 mV) was observed at the salt concentrations of 0.08 and 0.16 mm (Figure 2E). On the other hand, the bLg amyloid incubated with a higher Cu (II) salt concentration of 0.32 mm exhibited an overall neutral net charge for the first half of the measurement, indicating that a significant amount of metal ions had been sequestered by the bLg amyloid (Figure 2E). The acquired ζ‐potential values exhibited an increase‐decrease transition (Figure 2E). For both 0.16 and 0.32 mm Zn (II) salt‐incubated bLg amyloid samples, the net surface charge of the exhibited elevations followed by small decreases indicated that several metal ions were either chelated or electrostatically attracted by the bLg amyloid (Figure 2F). In contrast, a lower concentration of Zn (II) (0.064 mm) did not induce any significant changes in the bLg amyloid surface charge compared with the control (Figure 2F). Overall, the bLg amyloid exhibited a greater affinity for Zn^2+^ than for Cu^2+^, which is likely correlated with the higher stability of thiol–zinc than thiol–copper complexes. Similarly, bovine serum albumin (BSA) with a similar pI of 4.7 and one free cysteine formed amyloid biofilms and exhibited a greater efficiency for adsorbing Zn^2+^ than Cu^2+^.^[^ ^57^ ^]^

In addition, the maximum sequestration capacities of the bLg amyloid for copper and zinc ions were determined using a titration assay, where the metal ion concentrations were fixed at 10 mm, and the bLg amyloid concentration was varied from 0.001 to 5 mg mL^−1^. Both Cu and Zn elemental percentages determined using ICP‐MS exhibited a sigmoidal behavior with increasing bLg amyloid concentrations, and were fitted to sigmoidal four‐parameter logistic (4PL) curves. Specifically, the sequestration of both copper and zinc ions reached saturation levels for the bLg amyloid (≈0.1 mg mL^−1^) (Figure S4, Supporting Information).

Note that, while the bLg amyloid displayed a robust capacity for hosting metal ions, the association between the amyloid fibrils and the metal oxide nanoparticles themselves was minimal owing to their mutual charge repulsion and the energy penalty of adhering the metal oxide nanoparticles (30–50 nm in size) onto bLg amyloid fibrils with a large aspect ratio (≈1:1000, width to length). This was confirmed by a filtration assay, where minimal changes in the molar concentration of filtered nanoparticles (1 mm) were observed after 15 min and 4 h of pre‐incubation with the bLg amyloid (5 mg mL^−1^) (Figure S5, Supporting Information).

To further elucidate the mechanism of metal ion sequestration by bLg amyloids, we investigated the binding behavior of metal ions with bLg amyloids using DMD simulations. The amyloid‐forming segment ^117^LACQCL^122^ and the negatively charged segment ^41^VYVEELKPTPEG^52^ were selected to represent bLg fibrils because the fibril structure of the full‐length bLg protein is not currently available. Strong binding affinities for metal ions were observed at the Cys‐121 residue of bLg fibrils through the formation of coordination bonds (Figure 2G). Moreover, the negatively charged residues of the bLg fibrils exhibited high binding affinities for metal ions driven by electrostatic interactions (Figure 2H). In both cases, representative bLg fibrils provided metal ions with a string of binding sites along the fibril axis. As amyloid fibrils are usually micrometers in length and consist of hundreds or more peptides, a small number of amyloid fibrils can bind and immobilize metal ions of much higher molar concentrations to achieve the substoichiometric sequestration of metal ions. Thus, the robust immobilization of metal ions by the bLg amyloid was attributed to coordination bond formation and electrostatic interactions provided by the aligned Cys and Glu/Asp residues on the fibril surface.

### 2.3 Cytotoxic Responses to CuO and ZnO Nanoparticles and Biocompatibility of bLg Amyloid

**Figure 3:**
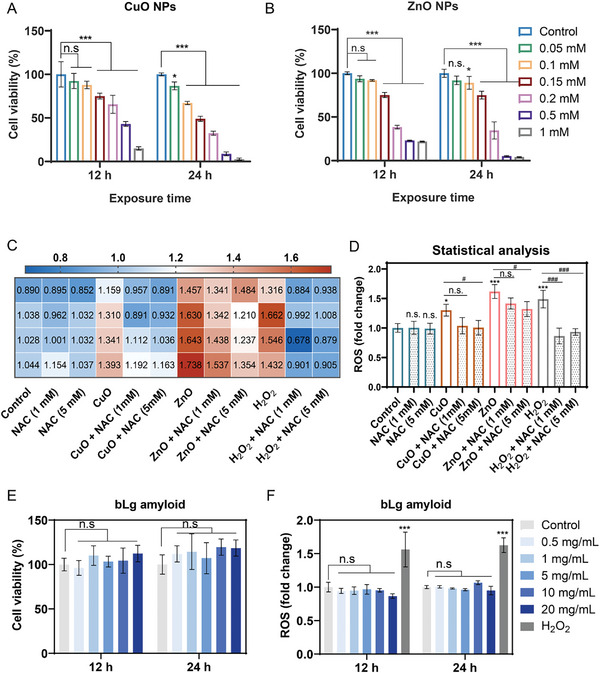
Cytotoxicity of CuO and ZnO nanoparticles and biocompatibility of the bLg amyloid to HUVECs upon exposure for 12 and 24 h. A,B) CCK‐8 assay was employed to measure the cell viability (%) of HUVECs upon exposure for 12 and 24 h to different concentrations of CuO (0.05–1 mm) (A) and ZnO nanoparticles (B) (0.05–1 mm). C) Fold change heatmap showing changes to ROS production for CuO (0.15 mm) and ZnO (0.15 mm) nanoparticle‐treated HUVECs after incubation for 4 h. The cells were either nontreated or pretreated with an ROS inhibitor, NAC (1 and 5 mm), for 2 h in DMEM medium. D) Statistical analysis of quadruplicate treated groups derived from panel (C). Data were analyzed via one‐way ANOVA followed by Tukey's post‐hoc test for multiple comparisons. E,F) Cell viability (%) (E) and ROS (F) assays performed in HUVECs after treatment for 12 and 24 h with bLg amyloid (0.5–20 mg mL^−1^) (n = 4). Positive control group cells for ROS assays were pretreated with 0.1 mm H_2_O_2_ in DMEM and incubated for 2 h. DCF fluorescence intensities (λ_ex_: 488 nm, λ_em_: 525 nm) were utilized for ROS quantification using a fluorescence microplate reader (n = 4). Data were analyzed via two‐way ANOVA followed by Tukey's post‐hoc test for multiple comparisons. All the data are depicted as mean ± SD and analyzed using GraphPad Prism. Statistically significant differences were considered as *p < 0.05, **p < 0.01, and ***p < 0.001.

Metal oxides, including CuO and ZnO nanoparticles, can enter organisms through skin contact, inhalation, and ingestion.^[^ ^58^, ^59^, ^60^ ^]^ In industrial environments, inhalation is the primary pathway of human exposure to metal oxide nanoparticles.^[^ ^61^ ^]^ Following inhalation, metal oxide nanoparticles, including CuO and ZnO, enter the alveolar region of the lungs, come into contact with the alveolar epithelium, and penetrate the lung epithelial barrier. This could promote their translocation into the bloodstream, thereby enabling direct interactions with the vascular endothelium and other organs to elicit biological and toxicological endpoints.^[^ ^62^, ^63^, ^64^ ^]^ The extent of toxic responses, including reactive oxygen species (ROS) production, cell adhesion loss, and mitochondrial dysfunction, can be elicited by metal oxide nanoparticles or synergistically by metal ions derived from particulates.^[^ ^61^ ^]^ Cellular exposure to metal ions has been shown to induce the upregulation of proteins responsible for metal homeostasis and detoxification, involving the overproduction of cysteine‐rich metallothionein molecules,^[^ ^65^, ^66^, ^67^ ^]^ as an adaptive response to metal stress in cells. This prompted us to further investigate whether the potent metal sequestration effect of the bLg amyloid could mimic the natural detoxifying mechanisms against the toxicity of metal oxide (CuO and ZnO) nanoparticles in endothelial HUVECs. As shown in **Figure** **3**A,B, both CuO and ZnO nanoparticles (0.05–1 mm) induced a dose‐dependent decline in HUVEC viability. Extending the exposure period of HUVECs to CuO and ZnO nanoparticles from 12 to 24 h led to an increase in cell mortality at all the nanoparticle concentrations by 6%–34% and 2%–18%, respectively (Figure 3A,B). The differentiation of toxicity mechanisms between trace metal species has been recently reported by Tsvetkov et al.,^[^ ^68^ ^]^ where copper toxicity was strongly correlated with mitochondrial lipoylated protein aggregation, resulting in proteotoxic stress and significant cell death.^[^ ^68^, ^69^ ^]^ The potential oxidative stress induced by CuO and ZnO nanoparticles at increasing concentrations (0.5–1 mm) was also investigated upon incubation with pre‐ or non‐treated HUVECs with an ROS inhibitor, N‐acetylcysteine (NAC) (Figure 3C,D). Both types of nanoparticles stimulated significant ROS production in untreated cells (Figure 3C,D, and Figure S6, Supporting Information), which was likely correlated with the direct interactions of the nanoparticles with oxidative organelles and redox‐active proteins. Furthermore, the soluble metal ions Cu^2+^ and Zn^2+^ produced by the nanoparticles have been shown to catalyze chemical reactions to generate free radicals. Among them, hydroxyl radicals (·OH) can be generated by the participation of Cu^2+^ in Fenton reactions, whereas abrupt superoxide radical (O_2_^·−^) formation can be catalyzed by both Cu^2+^ and Zn^2+^ through the autoxidation of biological molecules, such as lipids.^[^ ^70^ ^]^

The biocompatibility of the bLg amyloid (0.5–20 mg mL^−1^) was also tested in HUVECs. The results showed no notable cytotoxicity after 12 and 24 h of incubation, even at a relatively high concentration of 20 mg mL^−1^ (Figure 3E). To further evaluate the biocompatibility of the bLg amyloid, we examined whether its concentration range of 0.5–20 mg mL^−1^ could contribute to ROS production. A nonsignificant fold change in dichlorofluorescein (DCF) fluorescence was detected compared with that in the control (Figure 3F). This was consistent with other studies in which no apparent adverse effects on human cell lines (human corneal endothelial cells, human colon epithelial cells, or human epithelial cell lines) were elicited by digested/nondigested monomeric and amyloid forms of bLg.^[^ ^29^, ^32^, ^71^ ^]^

### 2.4 Alleviation of Cell Death Induced by CuO and ZnO Nanoparticles by bLg Amyloid

**Figure 4:**
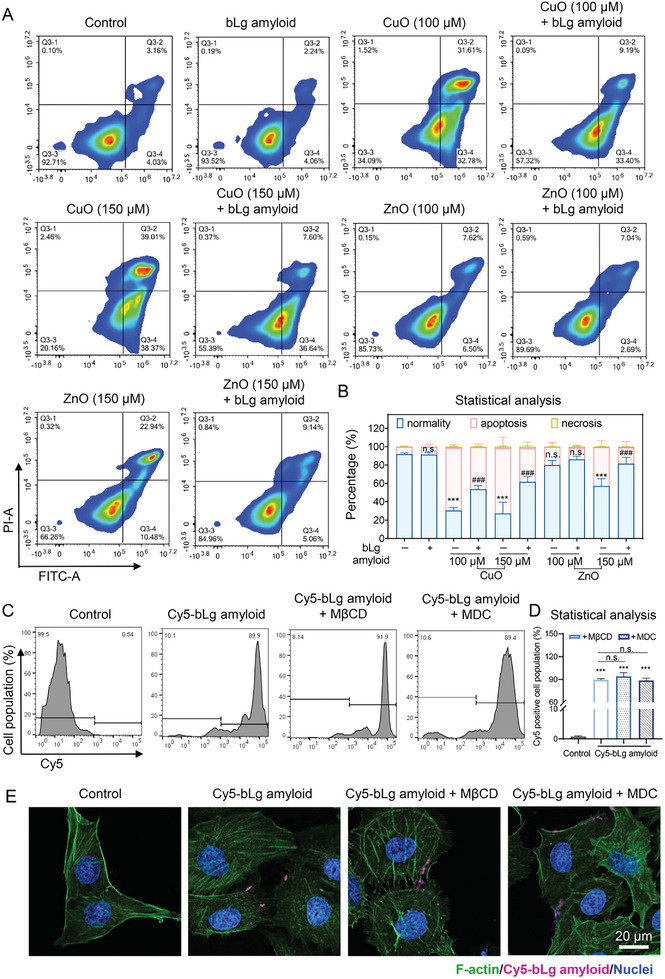
Rescue of HUVEC early/late cell apoptosis and cell necrosis induced by CuO and ZnO nanoparticles with bLg amyloid. A) Flow cytometry was used to classify necrotic and apoptotic HUVECs upon 24 h of exposure to CuO or ZnO nanoparticles (100, 150 µm) in the presence and absence of the bLg amyloid (5 mg mL^−1^). Apoptotic cells were labeled with Annexin V‐FITC, and necrotic cells were labeled with propidium iodide (Q3‐1: necrosis. Q3‐2: late apoptosis. Q3‐3: normal cells. Q3‐4: early apoptosis). B) Statistical evaluation of normal, apoptotic, and necrotic (%) cells in the control and treated cell groups presented in panel (A). Data were analyzed via two‐way ANOVA followed by Tukey's post‐hoc test for multiple comparisons (n = 3). C) Adhesion rate of the bLg amyloid on the surface of HUVECs in the presence and absence of endocytosis inhibitors. HUVECs were treated with MβCD (5 mm for 2 h) and MDC (10 µm for 1 h) before the bLg treatment. HUVECs were exposed to the bLg amyloid labeled with Cy5 (Cy5‐bLg) for 4 h, and Cy5 positive cells were detected by flow cytometry. D) Statistical analysis of Cy5 positive cells derived from the assay presented in panel (C). Data were analyzed via one‐way ANOVA followed by Tukey's post‐hoc test for multiple comparisons (n = 3). E) Confocal fluorescence images depicting the localization of the Cy5‐bLg amyloid (2.5 mg mL^−1^) in non‐/pre‐treated (MβCD/MDC) HUVECs upon exposure for 4 h. Channels: Nuclei (blue), Cy5‐bLg amyloid (purple), F‐actin (green). Scale bars: 20 µm, n = 3. All the data are depicted as mean ± SD. Significance between the treated and control groups is denoted as *p < 0.05, **p < 0.01, and ***p < 0.001. Similarly, ^#^ *p* < 0.05, ^##^ *p* < 0.01, and ^###^ *p* < 0.001 represented the significant differences between two treated groups.

Significant alterations to cell viability were observed following exposure to CuO and ZnO nanoparticles for 12 and 24 h at the concentrations of 100 and 150 µm, respectively (Figure 3A,B). The above nanoparticle concentrations were selected for subsequent cellular experiments. Flow cytometry analysis revealed that both concentrations of CuO nanoparticles induced marked cell mortality, which was primarily associated with early‐late apoptotic events (> 64%) in HUVECs (**Figure** **4**A). On the other hand, ZnO nanoparticles induced a significant cell mortality (> 33%) mainly related to apoptosis only at the concentration of 150 µm. The extent of necrosis was consistently lower (< 2.5%) than that of apoptosis in both nanoparticle treatments (Figure 4A). However, elevated necrosis was observed for CuO nanoparticles compared with ZnO nanoparticles (Figure 4A). The identified cell death mechanisms induced by metal oxide nanoparticles are consistent with previous findings,^[^ ^72^, ^73^, ^74^ ^]^ and are associated with microenvironmental changes owing to nanoparticle dissolution.^[^ ^58^, ^75^, ^76^, ^77^ ^]^ Therefore, based on the generally well‐established concept of metal‐promoted toxicity of ZnO and CuO nanoparticles, the sequestration of solubilized metal ions could serve as an effective strategy to mitigate the toxicities caused by metal oxide nanoparticles. To confirm this, HUVECs were exposed to increasing concentrations of CuCl_2_ (0, 200, and 400 µm) and ZnCl_2_ (0, 200, 400, and 500 µm) in the presence and absence of the bLg amyloid (5 mg mL^−1^), and their cell viabilities (%) were evaluated upon incubation for 24 h (Figures S7 and S8, Supporting Information). Exposure to 200 µm CuCl_2_ for 24 h induced a significant cytotoxicity to HUVECs, decreasing the viable cell population by ≈50% (Figure S7, Supporting Information), similar to the cytotoxicity effect observed for CuO nanoparticles at 150 µm (Figure 3A). In contrast, the bLg amyloid protected HUVECs at this CuCl_2_ dose, restoring their viable populations to normal levels (Figure S7, Supporting Information). On the other hand, the higher concentration of CuCl_2_ at 400 µm was observed to be extremely cytotoxic, and the bLg amyloid was unable to prevent cell death (> 95%) (Figure S7, Supporting Information), consistent with the saturation of the bLg amyloid to sequester free Cu ions (≈80% in the solution of 150 µm CuCl_2_ within 24 h, Figure S3, Supporting Information). In comparison with CuCl_2_, the cytotoxicity of ZnCl_2_ occurred at the higher doses of 400 and 500 µm, and the bLg amyloid again was effective in alleviating the metal‐induced cell death, by 31% and 51%, respectively (Figure S8, Supporting Information). This could be explained by the adsorption of Zn ions by the bLg amyloid, as its Zn‐binding capacity was close to 100% in a 150 µm ZnCl_2_ solution (Figure S3, Supporting Information), which is below the lethal level to HUVECs (Figure S8). Compared with ZnO nanoparticles (which caused >60% lethality at 200 µm) (Figure 3B), the observed greater resistance of HUVECs to elevated ZnCl_2_ concentrations (400 and 500 µm) may be attributed to the propensity of the ZnO nanoparticles to compromise cell membrane integrity and increase membrane permeability, leading to an influx of metal ions to facilitate cell death.^[^ ^78^ ^]^

To assess whether the affinity of the bLg amyloid for Cu^2+^ and Zn^2+^ could alleviate the metal oxide‐induced cytotoxicity, HUVECs were exposed to the combination of the bLg amyloid (5 mg mL^−1^) and CuO or ZnO nanoparticles (100 and 150 µm) for 24 h. Our flow cytometry analysis demonstrated that the bLg amyloid significantly reduced the proportions of early‐late apoptosis and necrosis induced by CuO and ZnO nanoparticles, as illustrated in Figure 4A,B. Specifically, the bLg amyloid alleviated the early‐late apoptosis induced by CuO and ZnO nanoparticles (150 µm) by 33% and 19%, respectively (Figure 4A). The efficacy of the bLg amyloid for reducing apoptosis and necrosis in HUVECs was also comparable against the lower concentration of 100 µm for the two types of nanoparticles (Figure 4A,B).

According to literature, the toxic effects caused by metal oxide nanoparticles are mainly attributed to the ions released by particle dissolution, and the “Trojan Horse effect,” which is attributed to the elevated dissolution of metal oxides upon nanoparticle cellular uptake.^[^ ^79^, ^80^ ^]^ The latter specific effect is responsible for the sharp increase in the concentration of metal ions in the intracellular environment, triggering a series of cytotoxic responses. Indeed, the experiments with HUVECs showed that metal‐ion release from CuO and ZnO nanoparticles was insufficient (≈50 µm Cu or Zn ions in DMEM, Figure 2A,B) to induce cell death (half‐lethal concentrations or LC_50_ ≈200 µm for CuCl_2_ and >500 µm for ZnCl_2_, Figures S7 and S8, Supporting Information). To elucidate whether the cellular uptake of CuO and ZnO nanoparticles played a role in their toxicity, we conducted an additional experiment using endocytosis inhibitors, methyl‐β‐cyclodextrin (MβCD) and monodansylcadaverine (MDC). Both MβCD and MDC are known for their distinct roles in the regulation of clathrin‐dependent endocytosis.^[^ ^81^ ^]^ Specifically, MβCD is involved in selectively extracting cholesterol from the plasma membrane, whereas MDC affects the alternate function of clathrin and clathrin‐coated vesicles. Herein, the exposure of 150 µm CuO and ZnO nanoparticles to noninhibitor‐treated HUVECs for 4 h induced apoptosis and necrosis in 11.5% and 6.5% of the cells, respectively (Figures S9 and S10, Supporting Information). On the other hand, MβCD rather than MDC significantly restored the ratio of normal cells in CuO and ZnO nanoparticle‐exposed groups to the levels of the control group (≈96%) (Figures S9 and S10, Supporting Information). These results indicated that CuO and ZnO nanomaterials were mostly taken up by HUVECs via caveolae‐mediated endocytosis, displaying a strong correlation between the toxic response and cellular internalization of CuO and ZnO nanoparticles (Figures S9 and S10, Supporting Information). Additionally, the bLg amyloid negligibly alleviated cell death (< 5%) after 4 h of exposure to CuO and ZnO nanomaterials (Figures S9 and S10, Supporting Information). As previously indicated, the detoxification effect of the bLg amyloid on nanoparticle‐treated HUVECs was notable after 24 h of co‐incubation (Figure 4A,B). The specific observed phenomenon was likely due to the capability of the bLg amyloid to not only sequester existing free copper or zinc ions (Figure S3, Supporting Information) but also continuously absorb solubilized copper or zinc ions from the nanomaterials, thus protecting HUVECs from cell death following a 24‐h exposure. This functionality effectively impedes the influx of additional metal ions or oxide nanoparticles into cells, thereby restricting the accumulation of copper or zinc within the intracellular environment. Subsequent ICP‐MS analysis indicated a significant reduction in the relative content of copper or zinc in the HUVECs treated with the bLg amyloid by 52% and 55%, respectively, compared with the HUVECs exposed to CuO or ZnO nanoparticles without the bLg amyloid (Figure S11, Supporting Information). The specific effect of the bLg amyloid contributed to the attenuation of cascade reactions initiated by excess intracellular metal ions, consequently alleviating the detrimental effects on the cellular environment (Figure 4A,B). In addition to HUVECs, consistent observations were made regarding the toxicity of CuO and ZnO nanoparticles and their mitigation by the bLg amyloid (Figures S12 and S13, Supporting Information), demonstrating a broader in vitro applicability of the remediation strategy.

To assess whether the bLg amyloid was taken up by HUVECs and contributed to the sequestration of Cu and Zn from CuO and ZnO nanoparticles, respectively, the bLg amyloid was labeled with Cy5 (Cy5‐bLg) and introduced into HUVECs for 4 h to further identify bLg cellular localization. A flow cytometric analysis indicated a significant proportion (93.5%) of Cy5‐positive HUVECs compared with the control, suggesting an association of amyloid fibrils with the cells (Figure 4C,D). Subsequently, we examined whether bLg amyloids could be internalized by HUVECs via endocytic uptake. The pretreatment of HUVECs with endocytosis inhibitors, MβCD and MDC, and further exposure of the cells to the Cy5‐bLg amyloid revealed that the introduction of the endocytosis inhibitors did not significantly reduce the proportion of Cy5‐bLg amyloid positive cells (Figure 4C,D). Consistent results were also obtained using confocal fluorescence microscopy, which revealed a high affinity of the Cy5‐bLg amyloid for the surface of HUVECs (Figure 4E and Figure S14, Supporting Information), likely owing to the hydrophobicity and large size of the fibrils, which promoted their association with amphiphilic cell membranes.

### 2.5 Delay in Lethality and Alleviation of Hepatotoxicity and Oxidative Stress Elicited by Metal Oxide Nanoparticles by bLg Amyloid In Vivo

**Figure 5:**
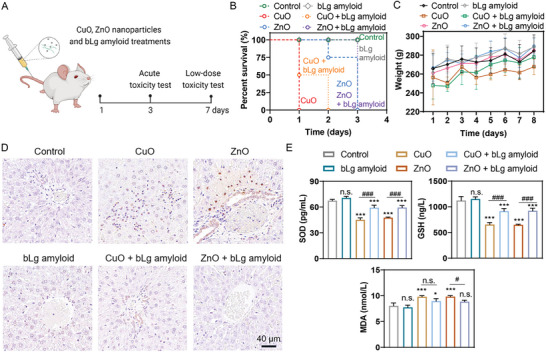
Prolongation of survival (%) and alleviation of hepatotoxicity and oxidative stress elicited by CuO and ZnO nanoparticles by bLg amyloid in a murine model. A) Schematic of the in vivo experimental design. Wistar rats were subjected to acute (40 mg kg^−1^) and low‐dose (10 mg kg^−1^) administration of CuO/ZnO nanoparticles through tail vein injection. Toxicity assays were evaluated over 3 and 7‐day exposure durations. B) Rat survival (%) monitored during a 4‐day post‐injection period. The rats were subjected to tail vein injection with CuO or ZnO nanoparticles (40 mg kg^−1^) in the presence and absence of bLg amyloid (20 mg kg^−1^) (n = 4 per group). C) Rats’ body weight (g) monitored during a 7‐day period upon low‐dose administration of CuO and ZnO nanoparticles (10 mg kg^−1^) with or without the bLg amyloid (20 mg kg^−1^) (n = 4 per group). D) TUNEL staining images of rat liver tissues upon low‐dose administration of CuO and ZnO nanoparticles (10 mg kg^−1^) with or without the bLg amyloid (20 mg kg^−1^) (n = 3 per group). All the images are of the same scale. Scale bar: 40 µm. E) Detection of oxidative stress indicators (SOD‐pg/mL, GSH‐ng/L, MDA‐nmol/L) in blood samples collected from the rats upon the low‐dose administration of CuO and ZnO nanoparticles (10 mg kg^−1^) with or without the bLg amyloid (20 mg kg^−1^) (n = 4 per group). Data are depicted as mean ± SD, and were analyzed via one‐way ANOVA followed by Tukey's post‐hoc test for multiple comparisons. *p < 0.05, **p < 0.01, and ***p < 0.001 represented significant differences compared with the control, #*p* < 0.05 and ###*p* < 0.001 represented significant differences between treatment groups, and n.s. represented no significant difference.

To determine whether the bLg amyloid could alleviate the toxic responses caused by metal oxide nanoparticles in vivo, Wistar rats were exposed to CuO or ZnO nanoparticles at acute (40 mg kg^−1^) and low doses (10 mg kg^−1^) via tail vein injection and monitored during 3‐ and 7‐day exposure periods, respectively (**Figure** **5**A). The biocompatibility of the bLg amyloid was initially assessed by monitoring rat body weight (g) during a 7‐day post‐administration period. All the bLg‐amyloid‐treated (20, 80, and 160 mg kg^−1^, n = 3) male Wistar rats attained or increased their mean body weight over 7 d and displayed growth similar to that of saline‐injected rats (n = 3) (Figure S15, Supporting Information). Additionally, key biochemical blood parameters, including white blood cell count and markers of hepatic and renal function, were normal in the bLg‐amyloid‐treated groups (20 and 80 mg kg^−1^) (Table S1, Supporting Information). The highest bLg amyloid dose of 160 mg kg^−1^ led to a decrease in the white blood cell count, whereas no statistically significant changes were observed in the other assessed parameters (Table S1, Supporting Information). The above results indicate the safety of such food‐extracted amyloids, consistent with studies performed with *C. elegans* and mouse models, where digested bLg amyloids elicited no apparent cytotoxicity or physiological abnormalities.^[^ ^29^ ^]^ To assess the antagonistic effect of the bLg amyloid on the acute or low‐dose toxicity of CuO and ZnO nanoparticles, the bLg amyloid was co‐administered to rats at a low dose of 20 mg kg^−1^. In the 3‐day acute toxicity experiment, ZnO nanoparticles (40 mg kg^−1^) induced a 75% mortality rate on the second day, whereas all the rats died on the third day (Figure 5B). The administration of CuO nanoparticles (40 mg kg^−1^) was observed to be more lethal, as all the rats died after one day of treatment (Figure 5B). Notably, after intervention with 20 mg kg^−1^ bLg amyloid, the mortality rate of the rats treated with CuO nanoparticles decreased from 100% to 50% on the first day. Moreover, the mortality rate of the rats treated with ZnO nanoparticles decreased from 25% to 0% on the second day (Figure 5B). Overall, these results suggest that the bLg functional amyloid serves as a potent substrate for sequestrating metal species and detoxifying copper and zinc, even at lethal concentrations of nanoparticle exposure.

In the low‐dose in vivo toxicity assays, the administered CuO and ZnO nanoparticle concentrations (10 mg kg^−1^) were based on the literature.^[^ ^74^ ^]^ The low‐dose administration of CuO nanoparticles in Wistar rats retarded the weight gain rate (4.4%) after 7 d of exposure compared with the control group (7.0%) (n = 4) (Figure 5C). In contrast, the rats treated with CuO nanoparticles in the presence of the bLg amyloid (20 mg kg^−1^) exhibited an elevated average growth rate (12.3%) compared with the saline‐injected control group (n = 4) over 7 d (Figure 5C). Normal weight gains (9.2%) were also observed in rats administered with low doses of ZnO nanoparticles (Figure 5C), indicating a difference in in vivo tolerance between CuO and ZnO nanoparticles.^[^ ^82^, ^83^ ^]^ The liver is a major organ involved in detoxification, and is mostly exposed to nanoparticles.^[^ ^84^ ^]^ Indeed, both CuO and ZnO nanoparticles induced hepatotoxic events related to apoptosis, as indicated by the positive terminal deoxynucleotidyl transferase dUTP nick‐end labeling (TUNEL)‐stained rat liver tissues presented in Figure 5D. In contrast, intervention with the bLg amyloid attenuated liver tissue apoptosis triggered by CuO and ZnO nanoparticles (Figure 5D). These results were consistent with the phenomena observed in the cellular assays (Figure 4A,B), suggesting that the metal sequestration capacity of the bLg amyloid also averted the adverse effects in vivo caused by metal oxide nanomaterials. As apoptosis is closely associated with oxidative stress,^[^ ^85^, ^86^ ^]^ representative oxidative stress marker levels (superoxide dismutase [SOD], reduced glutathione [GSH], and malondialdehyde [MDA]) were measured in rat blood samples after the administration of CuO or ZnO nanoparticles at 10 mg kg^−1^ with or without the bLg amyloid (20 mg kg^−1^) (Figure 5E). The results indicated that the administration of CuO and ZnO nanoparticles reduced the content of the antioxidant indicators SOD and GSH while increasing the content of the oxidant indicator MDA (Figure 5E). This imbalance in redox homeostasis is likely to mediate oxidative stress. Metal ions released from nanomaterials are known to bind to cell membrane receptors, interfere with enzyme activities, and disrupt mitochondrial function, resulting in immune dysfunction, an amplified inflammatory response, necrosis, and apoptosis.^[^ ^73^, ^87^, ^88^ ^]^ In contrast, the bLg amyloid protected against the decreases in SOD and GSH levels while mitigating the elevation of MDA levels, thereby counteracting the oxidative stress caused by CuO or ZnO nanoparticles (Figure 5E). Overall, the above results indicated that the metallothionein‐like antagonistic effect of the bLg amyloid on metal‐induced apoptosis and oxidative stress was likely due to efficient metal ion removal by the fibrils, eliminating the entry of dissolved metal ions into injured tissues or cells to alleviate adverse events downstream.

## 3 Conclusion

Over the past two decades, the field of NanoEHS has identified and elucidated a wide range of crucial nanotechnology‐derived environmental issues and their implications. Considerable research within the scope of NanoEHS has been dedicated to profiling the toxicity of metal oxide nanoparticles, which constitute a significant portion of the nanomaterial repertoire produced by industries and research laboratories. However, few viable strategies have been developed to effectively remediate the increasing environmental impacts of discharged nanomaterials. In this study, we demonstrated a scheme for mitigating the toxic burden exerted by CuO and ZnO nanoparticles in vitro and in vivo by repurposing whey‐protein‐derived functional bLg amyloid, which has been used for water purification and biomedical applications.^[^ ^28^, ^29^, ^31^ ^]^ Mechanistically, the protective effect of the functional bLg amyloid in the current study mimicked that of metallothionein, and was achieved through the efficient chelation and sequestration of the toxic ions released by the metal oxide nanoparticles, according to our quantitative ICP‐MS observations, zeta potential analysis, computer modeling, and cellular and in vivo assays (Figures 2, 3, 4, 5). Considering the wide‐ranging application and hence the rising environmental burden of metal oxide nanomaterials, and also considering the low cost and scale‐up capacity of functional protein amyloid synthesis, our study offered a crucial solution against the biological and environmental pitfalls of transition metal nanomaterials.

## 4 Experimental Section

### Preparation of Nanomaterial Stock Suspension

Nanoparticle bulk solutions were purchased from Aladdin Scientific China (ZnO) and XFNANO‐China (CuO). Nanoparticle stock suspensions (0.5 mg mL^−1^ CuO or 20 wt% ZnO) were sonicated for 5 min and further diluted in Milli‐Q water or DMEM (Gibco, USA) prior to each experiment.

### Dynamic Light Scattering and Zeta Potential Measurements

For the size distribution (Intensity%) and ζ‐potential (phase analysis light scattering method) measurements of nanomaterials, the CuO and ZnO nanoparticles were separately analyzed via the Zetasizer module (Brookhaven, USA) at room temperature. Electrophoretic light scattering was employed for measuring the ζ‐potential values of bLg amyloid (5 mg mL^−1^) samples in the presence and absence of CuCl_2_ (Aladdin Scientific) and ZnCl_2_ (Aladdin Scientific). After 10 min of incubation and centrifugation (20 000 *g* for 15 min), the pellets were collected and redispersed in 1.4 mL of Milli‐Q water. The solutions were placed in BI‐SCGO glass‐square cuvettes (Brookhaven, USA), which were compatible with an SREL solvent‐resistant electrode (Brookhaven, USA), and underwent 10 consecutive measurement acquisitions (three cycles per measurement) at a current below 2 mA.

### Preparation of bLg Solutions

Lyophilized powder of bLg from bovine milk was purchased from ACMEC Biochemical Technology (China). Protein purification from nonnative species and residual traces was performed based on a previous report by Jung et al.^[^ ^43^ ^]^ In brief, a concentrated protein solution (10 wt%, Milli‐Q water) was initially adjusted to acidic pH (4.6) with 1 m hydrochloric acid (HCl) solution. The solution was then centrifuged at 21,130 *g* for 15 min at 20 °C with a high‐speed centrifuge (Eppendorf AG, Germany). The supernatant was collected and adjusted to pH 2 with 1 m HCl solution, and subsequently filtered through a 0.22 µm Millipore filter (Biosharp, China). The filtered protein solution was transferred into dialysis membrane bags (MWCO 8000 Da, Coolaber Science & Technology) and dialyzed against pH‐2 Milli‐Q water at 4 °C for 4 h, and further against Milli‐Q water at 4 °C over 4 h. Finally, the purified bLg solution was freeze‐dried and stored at room temperature for subsequent experimental use.

### Preparation of bLg Amyloid Fibrils

Purified and lyophilized bLg powder was dissolved in Milli‐Q water at a concentration of 1 wt%. Upon centrifugation (10 800 *g* over 15 min) at 20 °C, the isolated supernatant was adjusted with HCl to pH 2 and filtered through a 0.45 µm Millipore filter. Mature bLg amyloid fibrils were obtained upon incubation at 90 °C for 6.5 h. SEC‐DAD was employed for the amyloid conversion rate (%) determination (detailed protocol see Supporting Information). For protein quantification, bLg amyloid solutions were purified using 50 kDa MWCO Amicon Ultra dialysis tubes (Millipore, Sigma Aldrich, USA). A Pierce bicinchoninic acid colorimetric assay (Absin, China) was used to quantify the total protein concentration following the commercial protocol. The purified bLg amyloid solutions were adjusted to a neutral pH for further use. For the synthesis of cyanine5‐labeled bLg (Cy5‐bLg) amyloid, 10 mg of the mono‐reactive succinimidyl dye ester of Cy5 (NHS‐Cy5, Pierce, USA) was completely dissolved in 1 mL of dimethyl sulfoxide. Subsequently, 4 mL of 1% (w/v) bLg amyloid solution was mixed with 616 µL of the NHS‐Cy5 solution and subjected to overnight incubation at 4 °C. The reaction mixture was then quenched and transferred to a dialysis bag (MWCO‐14 kDa, Viskase, USA). To facilitate the total removal of free NHS‐Cy5, the reaction mixture was dialyzed against 1 L of Milli‐Q water at 4 °C for 72 h. During dialysis, the water milieu was replaced every 18 h. The Cy5‐bLg amyloid solution was subsequently collected and dialyzed using a 50 kDa MWCO Amicon Ultra dialysis tube (Millipore). The purified Cy5‐bLg amyloid solution was finally stored at 4 °C for further use.

### Transmission Electron Microscopy

A small aliquot (10 µL) of each nanoparticle (1.25 mm) or bLg amyloid (0.1 mg mL^−1^) solution was dropped at the top of copper grids with a mesh size of 400 (Zhongjingkeyi Films Technology Co., Ltd). The grids were allowed to undergo a 30‐min incubation at room temperature, followed by the removal of excess liquid using a Whatman filter paper. The dried grids were subsequently treated with 5 µL of 3% aqueous solution of neutral‐pH‐adjusted phosphotungstic acid (Sigma Aldrich, USA) for 45 s. Intermediate and final grid drying including a single wash with Milli‐Q water (5 µL) was performed prior to imaging. TEM was performed using a Hitachi HT7800 electron microscope operated at 90 kV. Fibril width (W, nm) analysis was performed using ImageJ software by analyzing a large set of bLg fibrils (n = 200).

### Attenuated Total Reflection–FTIR Spectroscopy

ATR‐FTIR spectra within the wavenumber range of 1426–1700 cm^−1^ were obtained through an IRTracer‐100 from Shimadzu with a universal single‐reflection ATR accessory (PIKE MIRacle), a He–Ne laser, and an MCT detector (Hg–Cd–Te) maintained under liquid nitrogen cooling. The sample reservoirs were washed sequentially with Milli‐Q water and ethanol. Prior to each sample spectrum acquisition, an initial background scan was obtained using the specified operating data acquisition parameters (1426–1700 cm^−1^, 512 scans, 4 cm^−1^ resolution) and automatically subtracted using LabSolutions IR software. The bLg samples were lyophilized after incubation for 10 h at 2.5 mg mL^−1^ in a pH‐adjusted (7 and 9) aqueous environment. For acidic (pH 3) protein samples, previously HCl‐treated and purified monomer and amyloid powders (2.5 mg) were dissolved at 40 µL and small aliquots (5 µL) of each sample solution were added to the reservoir and allowed to air‐dry with the assistance of a heat‐gun. ATR‐FTIR spectra were acquired using the above data acquisition parameters and analyzed using a built‐in Peak Deconvolution application in Origin Software (OriginLab), following our previous methods.^[^ ^89^ ^]^

### Copper and Zinc Elemental Quantifications

ICP‐MS was employed to quantify the copper and zinc ions released from CuO and ZnO nanoparticles. CuO and ZnO nanoparticles were diluted to 0.25 mm (≈0.02 g L^−1^) in Milli‐Q water or DMEM medium. After 4, 8, 12, and 24 h of incubation, the suspensions were centrifuged at 20 000 *g* for 15 min, and the supernatants were collected. Aqueous solutions (0.25 mm) of CuCl_2_ and ZnCl_2_ were used as the controls. All the samples were analyzed using a Perkin Elmer NexION 2000 ICP‐MS spectrometer with ^103^Rh as the internal standard. The instrument parameters were set as follows: ICP RF power (1600 W), pulse voltage (1300 V); plasma gas flow (15 L min^−1^); auxiliary gas flow (1.2 L min^−1^); and nebulizer gas flow (0.86 L min^−1^).

### Evaluation of Metal Ion Adsorption by bLg Amyloid

To evaluate the adsorption capacity of metal ions, 0.25 mm CuO and ZnO nanoparticles were individually dispersed in DMEM medium and mixed with bLg amyloid (5 mg mL^−1^) in Eppendorf tubes. After 4, 8, 12, and 24 h of static incubation, the suspensions were vortexed and centrifuged at 20 000 *g* for 40 min. Similarly, 0.15 mm CuCl_2_ and ZnCl_2_ were individually incubated for 24 h in the presence of the bLg amyloid (5 mg mL^−1^) and centrifuged at 20 000 *g* for 40 min. For the sequestration saturation assay, 10 mm CuCl_2_ and ZnCl_2_ aqueous solutions were individually incubated for 1 h in the presence of the bLg amyloid (5, 0.5, 0.1, 0.01, and 0.001 mg mL^−1^) and centrifuged at 20 000 *g* for 40 min. The collected supernatants were examined using ICP‐MS following the above protocol (Experimental Section: Copper and Zinc Elemental Quantifications). All the samples were analyzed using Perkin Elmer NexION 2000 ICP‐MS spectrometer with ^103^Rh as the internal standard.

### Binding of Metal Oxide Nanoparticles with bLg Amyloid

The absorbance spectra of CuO and ZnO nanoparticles were initially acquired in the UV region (280–400 nm, step size 1 nm) across a range of molar concentrations (0–5 mm) in Milli‐Q water (100 µL, n = 3) using a multifunctional microplate reader (Tecan Spark, Switzerland). Standard curves were created based on the maximum absorbance band values (Abs_296 nm_‐CuO and Abs_362 nm_‐ZnO), and statistical analyses were performed using GraphPad via simple linear regression. To assess the interactions between the nanoparticles (CuO and ZnO) and the bLg amyloid, the dispersions of CuO and ZnO nanoparticles (1 mm) were incubated in the presence and absence of the bLg amyloid (5 mg mL^−1^) in Milli‐Q water (n = 3) at a volume of 0.8 mL. Following incubation for 15 min and 4 h, the mixtures were vortexed for 1 min and gently filtered using nylon membrane filters (pore size 0.45 µm, diameter 25 mm, Jin Teng Technology Co.) to isolate the unbound nanoparticles from the amyloid species. The collected filtrates were subsequently vortexed for 1 min, and the Abs_296,362 nm_ values of 100 µL filtrate solutions (n = 3) were acquired in 96‐well clear bottom plates (Corning, USA). The concentration (mM) values were determined by normalization between the blank sample Abs_296,362 nm_ values used for the standard curves and filtration assay. Nonfiltered nanoparticle dispersions (1 mm) in Milli‐Q water served as the reference standards.

### Discrete Molecular Dynamics Simulations

DMD was a special molecular dynamics algorithm in which continuous interaction potentials in classical molecular dynamics were replaced by optimized stepwise functions.^[^ ^90^ ^]^ Atoms in DMD move with constant velocities until they meet at the energy step, where the velocities were updated according to conservation laws. Thus, the dynamics of atoms in the DMD were dictated by iteratively updating the colliding atoms, predicting new collisions, and finding the next collision using quick‐sort algorithms.^[^ ^91^ ^]^ Compared with classical molecular dynamics, the computational efficiency and sampling quality of DMD were significantly enhanced, thereby enabling theoretical studies on protein folding, amyloid peptide aggregation, and nanoparticle–protein interactions. This study examined the binding of metal ions to bLg amyloid fibrils in silico. To model bLg amyloid fibrils that were structurally unknown and likely heterogeneous, two bLg‐amyloid‐forming segments, ^117^LACQCL^122^ and ^41^VYVEELKPTPEG^52^ containing ion‐binding cysteines and acidic amino acids, were selected from fragments identified by a mass spectroscopy study of protease‐resistant bLg amyloid fibril cores.^[^ ^92^ ^]^ The corresponding fibrillar structures of both sequences were predicted using Fibpredictor^[^ ^93^ ^]^ and relaxed in the DMD simulations for 50 ns. For the docking of metal ions with the bLg amyloid, we assigned attractive potentials between the metal ions and cysteine atoms to model coordination bonds.^[^ ^94^ ^]^ The potentials were determined by calculating the histograms of the atomic distances in structures containing metal ions available in the PDB databank, as in a previous study.^[^ ^94^ ^]^

### Cell Culture

HUVECs were obtained from the ATCC. Hep 3B cells were purchased from Pricella Life Science and Technology. According to standard culture conditions (37 °C, 5% CO_2_), HUVECs were maintained in DMEM medium and Hep 3B were cultured in MEM, both containing 1% penicillin‐streptomycin and 10% fetal bovine serum (Gibco, USA). The cells were passaged once they reached 80% confluence. The number of passages was less than 10.

### Cell Viability Assay

A CCK‐8 assay kit (Yeasen, China) was used for cell viability analysis. HUVECs (8 × 10^3^ cells per well) or Hep 3B cells (1 × 10^4^ cells per well) were seeded in 96‐well plates (Corning, USA) and cultured at 37 °C for 4–6 h. The cells were then exposed to varying concentrations of bLg amyloid, CuO, or ZnO nanoparticles, and the incubation period was extended to either 12 or 24 h. The culture medium was removed, and the cells were washed three times with phosphate‐buffered saline (PBS) (Gibco, USA). Subsequently, the cells on each well were subjected to 100 µL serum‐free medium containing 10 µL of the CCK‐8 reagent. The cells were then incubated in an incubator at 37 °C for 1 h. Following incubation, the absorbance at 450 nm was measured and recorded using a multifunctional microplate reader (Tecan Spark, Switzerland).

### Reactive Oxygen Species Detection

HUVECs were seeded at a density of 8 × 10^3^ cells per well in 96‐well plates and exposed to CuO and ZnO nanoparticles and different concentrations of bLg amyloid (0.5, 1, 5, 10, and 20 mg mL^−1^). For Hep 3B cells, 1 × 10^4^ cells per well were cultured in 96‐well plates and treated with the bLg amyloid (0.5, 1, 5, 10, or 20 mg mL^−1^). Cells treated with H_2_O_2_ (0.1, 2 h) were used as positive controls. Cells were pre‐incubated with NAC (1 and 5 mm, 2 h) to test the ROS generation. The cells were then treated with the sample groups, and the cell culture medium was removed. The cells were washed twice with PBS. 2’,7’‐dichlorodihydrofluorescein diacetate (DCFH_2_‐DA) fluorescent dye (5 µm) was applied to the cells and incubated for 30 min in dark. Excess dye was removed, and the cells were washed twice with PBS. The fluorescence intensity of the intracellular ROS, centered at 525 nm and excited at 488 nm, was determined using a multifunctional microplate reader (Tecan Spark, Switzerland).

### Flow Cytometry

HUVECs were seeded in 6‐well plates (2 × 10^6^ cells per well) and allowed to grow for 6 h. For the apoptosis and necrosis detection assays, the cells were treated with the bLg amyloid and CuO or ZnO nanoparticles and allowed to incubate (37 °C, 5% CO_2_) for 24 h. The cells were washed with PBS and collected by digestion with ethylenediaminetetraacetic acid‐free trypsin (Beyotime, China). After two additional washes with PBS (800 *g*, 5 min, 25 °C), each cell sample was collected and resuspended in 400 µL of binding buffer. Subsequently, 5 µL of Annexin V‐FITC and 5 µL of propidium iodide (BD, USA) were sequentially added to each cell sample. The mixture was then incubated for 15 min in the dark at room temperature. Subsequently, the cells were analyzed using flow cytometry (Agilent, USA). For accessing the association of HUVECs with the Cy5‐bLg amyloid, the cells were treated with clathrin‐dependent endocytosis inhibitors, MβCD (Selleck, USA) and MDC (Selleck, USA). Specifically, HUVECs were pretreated with MβCD (5 mm) for 2 h and MDC (10 µm) for 1 h, washed twice with PBS, and then incubated with DMEM medium containing Cy5‐bLg amyloid (5 mg mL^−1^) for 4 h. The cells were collected using trypsin in the absence of ethylenediaminetetraacetic acid (EDTA) (Beyotime, China) and resuspended in 500 µL PBS solution for flow cytometry.

### Elemental Detection of Copper and Zinc in HUVECs

HUVECs (3 × 10^6^ cells per well) were seeded in 6‐well plates and were allowed to incubate for 6 h (37 °C, 5% CO_2_) to form adherent cultures. The HUVECs were further treated with CuO or ZnO nanoparticles (150 µm) in the presence and absence of the bLg amyloid (5 mg mL^−1^) in DMEM medium and allowed to incubate for 4 h (37 °C, 5% CO_2_). The cellular milieus was subsequently discarded, and the cells were washed three times with PBS to remove excess CuO and ZnO nanomaterials. HUVECs were digested with EDTA‐free trypsin, and HUVEC pellets were collected upon centrifugation at 800 *g* for 5 min (25 °C) and supernatant removal. A hydrochloric acid solution (1 mL, 1% v/v) was added to the cells, following sonication for 1.5 h and incubation for 16 h at room temperature. The digested cells were centrifuged at 20 000 *g* for 40 min (25 °C), and the collected supernatants were finally diluted five times with Milli‐Q water so that their elemental (Cu, Zn) content could be determined through ICP‐MS.

### Confocal Fluorescence Microscopy

HUVECs (7 × 10^4^ cells per well) were seeded in confocal dishes (35 mm; Nest Biotechnology) and cultured for 24 h. The cells were then treated with the Cy5‐bLg amyloid (2.5 mg mL^−1^) for 4 h. Upon complete removal of the culture medium, the cells were washed twice with PBS and fixed in 4% paraformaldehyde (Solarbio, China) for 20 min. After two washes with PBS, the cells were treated with PBS containing 0.1% Triton X‐100 and washed three times (5 min each). Subsequently, Actin‐Tracker Green solution (Beyotime, China) was applied to the cells in a confocal dish at a dilution of 1:50 in 5% BSA. The cells were incubated in the dark for 30 min. After a PBS wash, the cells were stained with a 4',6‐diamidino‐2‐phenylindole (DAPI) solution (Solarbio, China) at a concentration of 10 µg mL^−1^ for 5 min. Confocal fluorescence microscopy images were acquired using a 20× objective with an FITC filter set (excitation: 488 nm, emission: 540 nm) and a Cy5/deep red filter (excitation: 652 nm, emission: 669 nm) on a confocal fluorescence microscope (Nikon Eclipse Ti2‐E).

### In Vivo Experiments with Animal Model

All the in vivo experiments were supported by the IACUC (Institutional Animal Care and Use Committee) at the Center for Nano‐Biology Safety Evaluation and Research, GBA National Institute for Nanotechnology Innovation, Guangzhou, China (IACUC number: IA‐T2250710182‐02). Male Wistar rats (9 weeks old) were purchased from Shanghai SLAC (Shanghai, China). The rats were kept in cages (n = 4 per cage) under specific pathogen‐free conditions, with free access to food and water under a controlled temperature (22–24 °C) and equal light/dark cycle (12:12 h). For the 3‐day acute toxicity monitoring experiments, the rats were randomly divided into six groups (n = 4 per group), and CuO and ZnO nanoparticles (40 mg kg^−1^) in the presence and absence of the bLg amyloid (20 mg kg^−1^) were dispersed in 1 mL of physiological saline per group. The rats were subjected to tail vein injection, and the negative control groups were administered 1 mL of physiological saline. For the 7‐day low‐dose toxicity monitoring experiments, animal injections and groupings were designed similarly. Specifically, the concentrations of the CuO and ZnO nanoparticles were reduced to 10 mg kg^−1^, whereas the bLg amyloid concentration remained unchanged (20 mg kg^−1^). The body weights (g) of the rats were recorded daily. At the end of the monitoring experiment, the rats were completely anesthetized with carbon dioxide and euthanized by cervical dislocation. The liver was dissected, and their tissues were extracted and kept at −80 °C for subsequent experiments.

### Oxidative Stress Marker Analysis

On the seventh day following administration, rat blood samples were collected and subsequently centrifuged at 1503 *g* and 4 °C for 15 min. The SOD, GSH, and MDA levels in the blood were quantified using commercially available detection kits sourced from Elabscience, China, following the manufacturer's protocol. Absorbance (*A*) measurements for determining each indicator's content (SOD:*A*_450nm_, GSH:*A*_420nm_, MDA:*A*_532nm_) were performed using a microplate reader (Tecan Spark, Switzerland).

### Terminal Deoxynucleotidyl Transferase dUTP Nick End Labeling Assay

Rat liver tissues were fixed in 10% neutral buffered formalin (ACMEC, China). Following fixation, the tissues were embedded in paraffin (Leica, Germany) and sliced into 5‐µm‐thick sections. The sections were affixed to glass slides and dried in an oven at 65 °C for 1 h to eliminate moisture. Deparaffinization was achieved by immersing the samples in xylene (Sigma‐Aldrich, USA) for 5–10 min, followed by repeated deparaffinization with fresh xylene. Subsequently, the samples were subjected to a series of alcohol washes with varying concentrations (anhydrous ethanol, 5 min; 90% v/v ethanol, 2 min; 70% v/v ethanol, 2 min). A colorimetric TUNEL apoptosis assay kit (Beyotime, China) was used for subsequent staining. A 2‐min rinse with cold water was performed, and then, 20 µg mL^−1^ DNase‐free protease K (Beyotime, China) was added to the samples, which were then incubated at 37 °C for 20 min. After three washes with PBS, the samples were treated with PBS containing 3% hydrogen peroxide (H_2_O_2_) at room temperature for 20 min. Subsequently, the samples were washed three times with PBS for 2 min each, and 50 µL of biotin labeling solution (Beyotime, China) was added dropwise to the samples. After 1 h of incubation in the dark at 37 °C, the samples were rinsed three times with PBS and allowed to stand for 2 min per wash. Subsequently, 50 µL of streptavidin horseradish peroxidase working solution (Beyotime, China) was added to the samples, and they were allowed to incubate for 30 min, followed by three washes of PBS. 3,3′‐diaminobenzidine (DAB) solution was applied to cover the samples for 15 min, followed by three washes with PBS (2 min per wash). After staining with hematoxylin for 2 min, the samples were dehydrated using an alcohol gradient. The samples were sealed and dried prior to observation using an Axio Vert.A1 fluorescence microscope (ZEISS, Germany).

### Statistical Analysis

Data were analyzed using GraphPad Prism software and presented as the mean ± standard deviation (SD). Student's *t*‐test, one‐way analysis of variance (ANOVA), or two‐way ANOVA were employed to evaluate statistically significant differences between sample groups, followed by Tukey's post‐hoc tests for multiple comparisons. Statistically significant differences were considered as *p < 0.05, **p < 0.01, and ***p < 0.001 compared with the control group, and #*p* < 0.05, ##*p* < 0.01, and ###*p* < 0.001 represented significant differences between the treatment groups. Nonsignificant differences were represented as n.s.

## Conflict of Interest

The authors declare no conflict of interest.

## Author Contributions

Y.W. and X.L. contributed equally to this work. P.C.K. and M.M. conceived the study. P.C.K. and Y.W. designed the experiments. Y.W., X.L., and Y.L. performed the bLg purification and amyloid synthesis. N.A., Y.W., and X.L. conducted the TEM, DLS, and zeta potential measurements of the metal oxide nanoparticles. X.L. and Y.W. performed the ICP‐MS characterization of metal oxide ion release and sequestration (including saturation) by the bLg amyloid, and the SEC characterization of the bLg monomer to amyloid conversion. N.A. performed the FTIR secondary structural analyses of bLg monomers and amyloids and a filtration assay for amyloid‐nanoparticle association. Y.W. performed in vitro cell viability, ROS, and flow cytometry assays. Y.W. performed the in vivo toxicity and remediation assays assisted by F.H., X.Y., and G.P. H.T. and F.D. conducted DMD simulations and analyses. N.A., Y.W., and P.C.K. wrote the manuscript, and G.P. and M.M. provided critical comments. All the authors have discussed and agreed upon the presentation of the manuscript.

## Supporting information

Supporting Information

Supporting Information

## Data Availability

The data that support the findings of this study are available in the supplementary material of this article.
