# Supplementary material for: Remediation of Metal Oxide Nanotoxicity with a Functional Amyloid
Source: Adv Sci (Weinh). 2024 Apr 6;11(23):2310314. doi: 10.1002/advs.202310314 (PMC11187920; doi:10.1002/advs.202310314)
Supplement: Supplementary file 1 — Supporting Information [file ADVS-11-2310314-s001.pdf]

ADVANCED SCIENCE
Open Access

Supporting Information

for *Adv. Sci.*, DOI 10.1002/advs.202310314

Remediation of Metal Oxide Nanotoxicity with a Functional Amyloid

Yue Wang, Xiufang Liang, Nicholas Andrikopoulos, Huayuan Tang, Fei He, Xiang Yin, Yuhuan Li, Feng Ding, Guotao Peng*, Monika Mortimer* and Pu Chun Ke**

Supporting Information

Remediation of Metal Oxide Nanotoxicity with a Functional Amyloid

Yue Wang,[1,2†] Xiufang Liang,[1,2†] Nicholas Andrikopoulos,[2,3] Huayuan Tang,[4,5] Fei He,[6] Xiang Yin,[6] Yuhuan Li,[3,7] Feng Ding,[5] Guotao Peng,[6*] Monika Mortimer,[8*] Pu Chun Ke[2,3*]*

Yue Wang, Xiufang Liang
School of Biomedical Sciences and Engineering, Guangzhou International Campus, South China University of Technology, Guangzhou 510006, China
Nanomedicine Center, The Great Bay Area National Institute for Nanotechnology Innovation, 136 Kaiyuan Avenue, Guangzhou 510700, China

Dr. Nicholas Andrikopoulos, Prof. Pu Chun Ke
Nanomedicine Center, The Great Bay Area National Institute for Nanotechnology Innovation, 136 Kaiyuan Avenue, Guangzhou 510700, China
Drug Delivery, Disposition and Dynamics, Monash Institute of Pharmaceutical Sciences, Monash University, 381 Royal Parade, Parkville, VIC 3052, Australia

Dr. Huayuan Tang
Department of Engineering Mechanics, Hohai University, Nanjing 211100, China
Department of Physics and Astronomy, Clemson University, Clemson, SC 29634, USA

Prof. Feng Ding
Department of Physics and Astronomy, Clemson University, Clemson, SC 29634, USA

Fei He, Xiang Yin, Dr. Guotao Peng
College of Environmental Science and Engineering, Key Laboratory of Yangtze River Water Environment, Tongji University, 1239 Siping Road, Shanghai 200092, China

Prof. Yuhuan Li
Drug Delivery, Disposition and Dynamics, Monash Institute of Pharmaceutical Sciences, Monash University, 381 Royal Parade, Parkville, VIC 3052, Australia
Liver Cancer Institute, Zhongshan Hospital Key Laboratory of Carcinogenesis and Cancer Invasion, Ministry of Education, Fudan University, Shanghai 200032, China

Prof. Monika Mortimer

Laboratory of Environmental Toxicology, National Institute of Chemical Physics and Biophysics, Akadeemia tee 23, 12618 Tallinn, Estonia

E-mail: Dr. Nicholas Andrikopoulos, nicholas.andrikopoulos@monash.edu; Dr. Guotao Peng, guotaopeng@tongji.edu.cn; Prof. Monika Mortimer, monika.mortimer@kbfi.ee. Prof. Pu Chun Ke, pu-chun.ke@monash.edu.

† **These authors contributed equally.**

Size exclusion chromatography-diode array detection (SEC-DAD): bLg amyloid samples were prepared according to the standard protocols of Veerman et al.[1] and Jung et al.[2] Additional information can be found in the **Experimental Section**. *Preparation of bLg amyloid fibrils*. Upon incubation at 90 °C for 6.5 h, bLg amyloid solutions underwent a dilution with Milli-Q and a pH adjustment at 4.8. Subsequent centrifugation at 20,000 *g* for 15 min at 20 °C yielded pelleted samples. Monomer supernatants were collected and analyzed using a 1290 Infinity II UPLC system (Agilent, USA), adopting the following instrument parameters: sample manager thermostat set at 15 °C, column oven thermostat set at 30 °C, and injection volume of 5 μL. An AdvanceBio SEC column (4.6×150 mm, particle size 1.9 μm, pore size 200 Å) (Agilent, USA), equilibrated with PBS, was running at a flow rate of 0.3 mL min^{-1}. The column was connected to a DAD detector (λ=280 nm) for online SEC/DAD analysis. The amyloid conversion rate was determined by analyzing the intensity peak height (mAU) of the bLg monomer and amyloid samples (n=2).

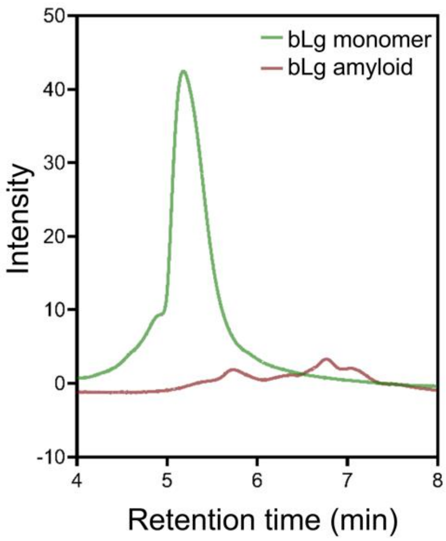

Samples	Peak height (mAU)	Amyloid conversion (%)
bLg monomer	33.626 ± 1.546	-
bLg amyloid	1.711 ± 0.0184	94.9

Figure S1. bLg amyloid conversion rate analysis. Representative SEC chromatographs of the monomers and bLg amyloid fibrils collected in supernatants. The SEC chromatograph conversion rate was determined by analyzing the intensity peak heights (mAU) between the bLg monomer and amyloid samples. Data are depicted as mean ± SD (n=2).

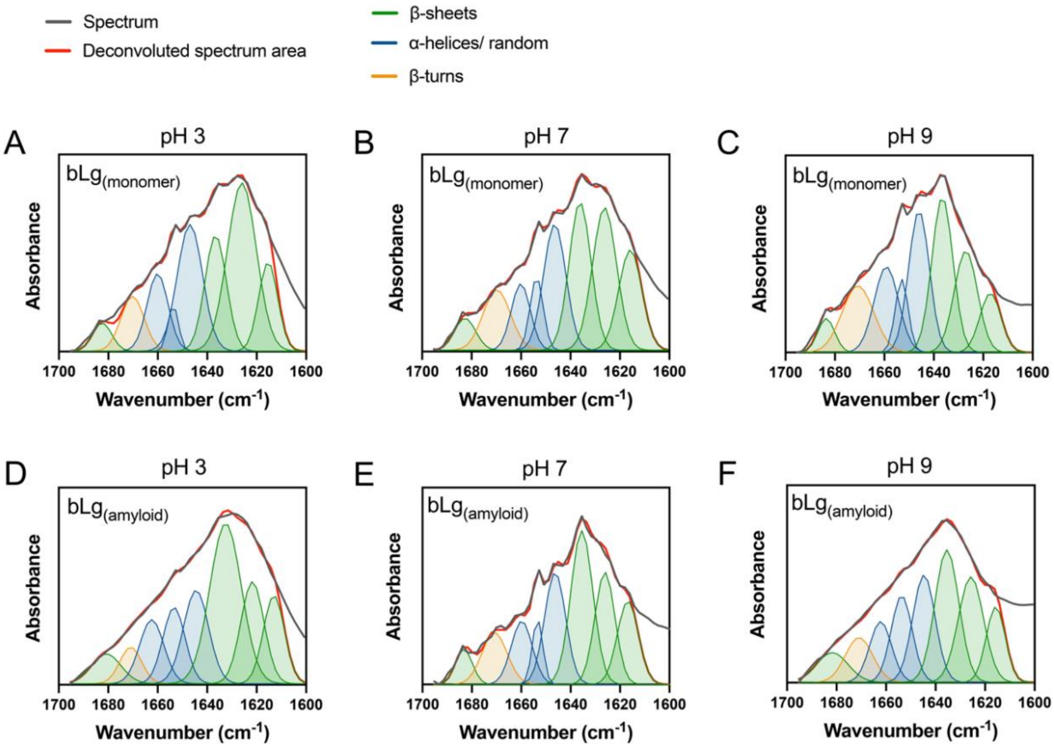

Figure S2. Secondary structure analysis of the bLg monomer and amyloid species within a pH range (3, 7, and 9) determined using ATR-FTIR. The amide I band spectra (1600–1700 cm^{-1}) were obtained from **Figure 1F&G** and analyzed through a peak convolution tool (OriginLab). Each deconvoluted peak was assigned based on the corresponding wavenumber region for each type of protein conformation (β-sheets: green, α-helices/random: blue, β-turns: orange).[3]

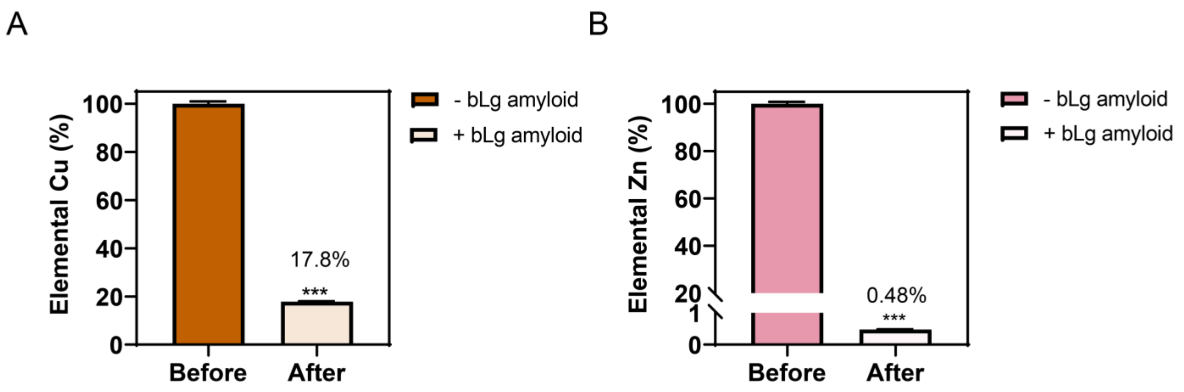

Figure S3. ICP-MS elemental analysis of metal ion sequestration by the bLg amyloid before and after incubation with copper (A) or zinc (B) salt in Milli-Q water. $CuCl_2$ or $ZnCl_2$ solutions (150 μM) were incubated with the bLg amyloid (5 mg/mL) for 24 h. Statistical analysis through the conduction of t-tests (*p<0.05, **p<0.01, and ***p<0.001) was used for the evaluation of statistical differences between the two groups. Data are depicted as mean ± SD (n=3).

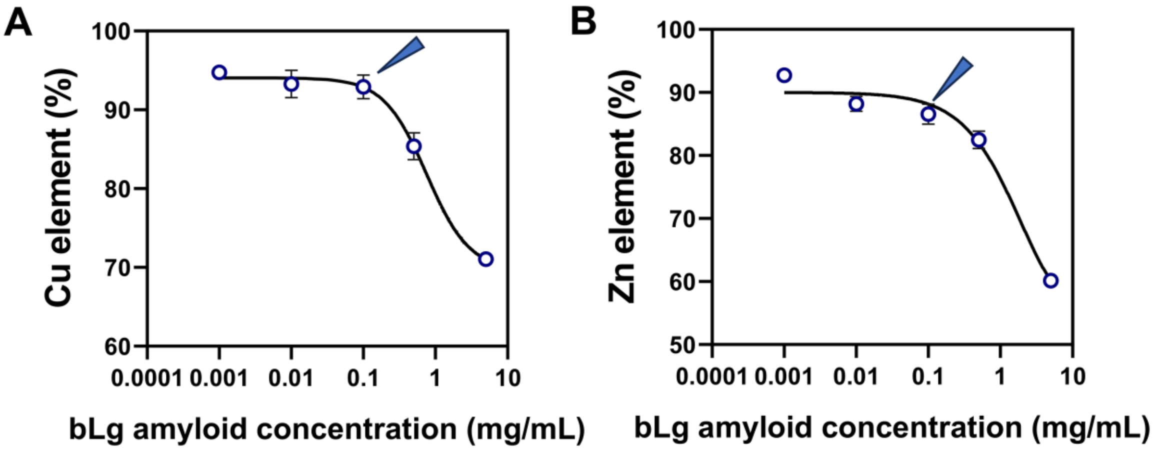

Figure S4. Titration assay performed through ICP-MS elemental analysis depicting the saturation levels of copper and zinc ions sequestered by the bLg amyloid upon 1 h of incubation. (A) $CuCl_2$ or (B) $ZnCl_2$ solutions (10 mM) were incubated with increasing bLg amyloid concentrations (5, 0.5, 0.1, 0.01, and 0.001 mg/mL) for 1 h in Milli-Q water (n=3). Element (%) values were fitted with sigmoidal 4PL curves via GraphPad Prism. Data are depicted as mean ± SD (n=3).

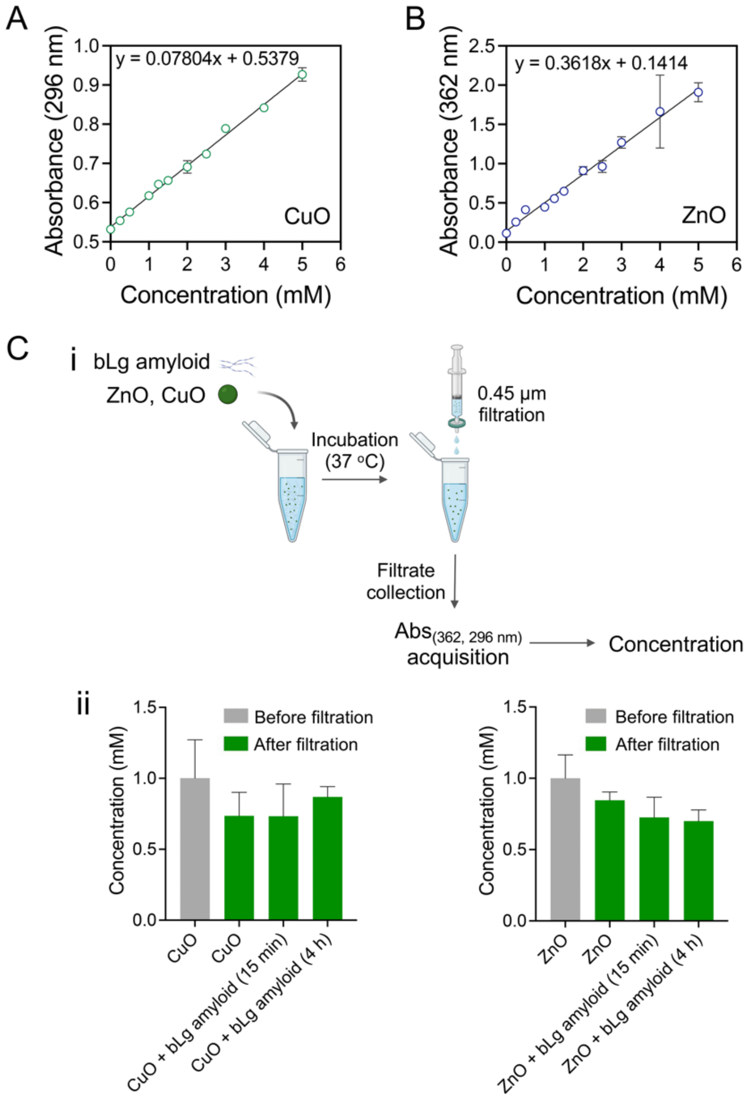

Figure S5. Determination of unbound CuO and ZnO nanoparticles upon co-incubation for 15 min and 4 h with the bLg amyloid. (A, B) Standard curves for CuO (A) and ZnO (B) nanoparticles in Milli-Q water derived from the maximum absorbance values (n=3) at their respective wavelengths (296 nm for CuO,[4] 362 nm for ZnO[5]) plotted against their molar concentrations (0, 0.25, 0.5, 1, 1.25, 1.5, 2, 2.5, 3, 4, and 5 mM), showcasing the linear relationship for quantitative analysis. (C) (i) Schematic of the experimental procedures. CuO and ZnO nanoparticles (1 mM) were incubated with and without the bLg amyloid (5 mg/mL) in Milli-Q water. Following incubation (37 °C) and filtration through 0.45 µm Nylon membrane filters, the absorbance values at 296 nm and 362 nm were measured to quantify the unbound nanoparticles. (ii) Concentration of the nanoparticle suspensions in the presence and absence of the bLg amyloid (5 mg/mL) in Milli-Q water. Nonsignificant changes in the molar concentration of filtered nanoparticles were observed after pre-incubation with and without the bLg amyloid. Data are depicted as mean ± SD and analyzed via one-way ANOVA followed by

Tukey's post-hoc test for multiple comparisons (n=3). Statistically significant differences between the treated groups were considered as $p<0.05$. Unfiltered nanoparticle suspensions at 1 mM were utilized as the reference standards.

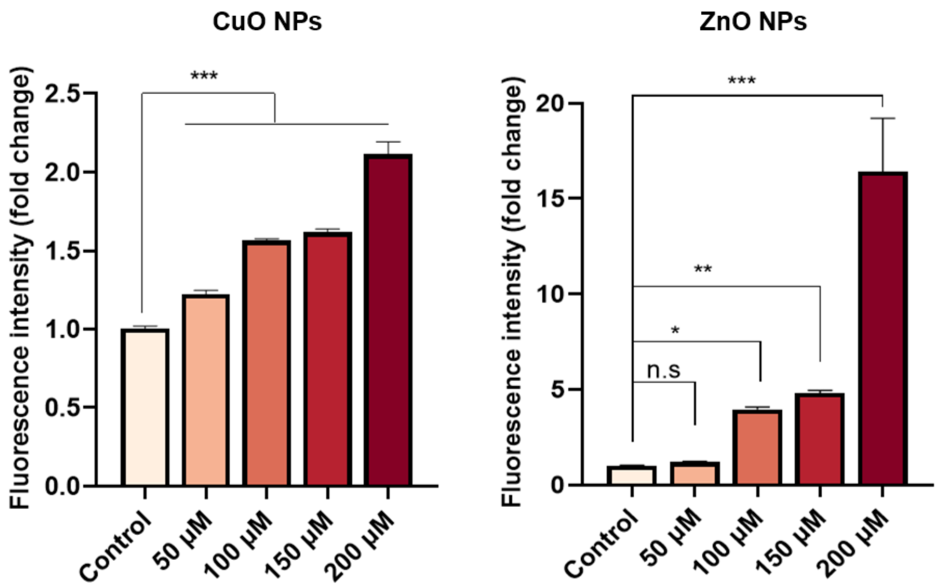

Figure S6. Reactive oxygen species (ROS) detection in HUVECs, upon treatment for 24 h with CuO or ZnO nanoparticles (50, 100, 150, and 200 μM). DCF fluorescence intensity (λ_{ex}: 488 nm, λ_{em}: 525 nm) was quantified using a fluorescence microplate reader. Data are depicted as mean ± SD and analyzed via one-way ANOVA followed by Tukey's post-hoc test for multiple comparisons (n=4). Statistically significant differences between CuO- or ZnO-nanoparticle-treated groups and control groups were considered as *$p<0.05$, **$p<0.01$, and ***$p<0.001$.

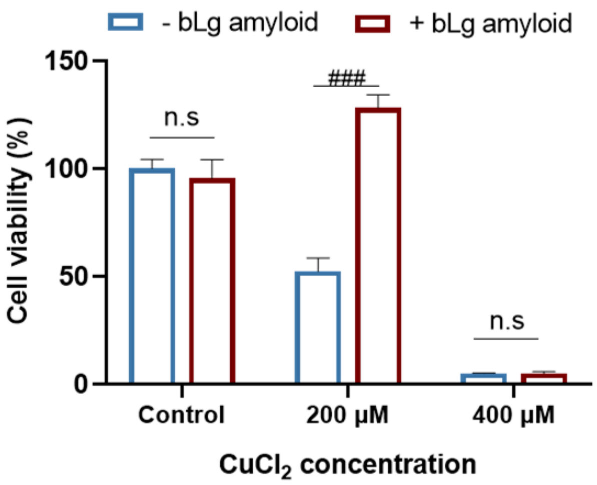

Figure S7. Cell death induced by copper ions alleviated by bLg amyloid. HUVECs viability following a $CuCl_2$ (0, 200, 400 μM) treatment with or without the bLg amyloid (5 mg/mL) for 24 h. Data are presented as mean ± SD (n=4) and analyzed via two-way ANOVA followed by Tukey's post-hoc test for multiple comparisons. Statistically significant differences between equally concentrated $CuCl_2$ sample groups were considered as $^{\#}p<0.05$, $^{\#\#}p<0.01$, and $^{\#\#\#}p<0.001$ compared with the bLg amyloid treatment group.

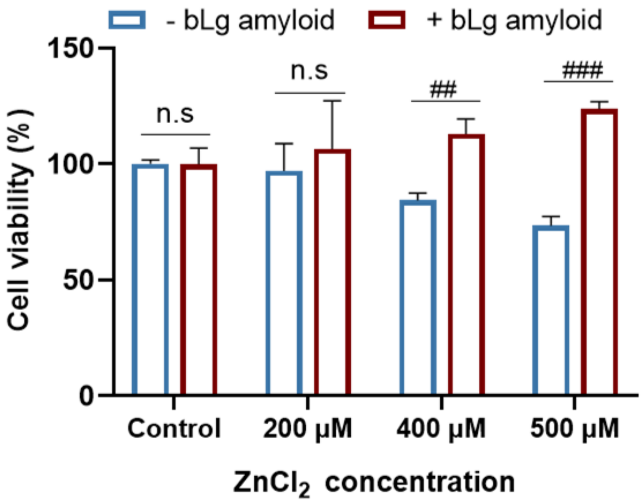

Figure S8. Cell death caused by zinc ions alleviated by bLg amyloid. HUVECs viability following a treatment with $ZnCl_2$ (0, 200, 400, and 500 μM) for 24 h in the presence and absence of the bLg amyloid (5 mg/mL). Data are presented as mean ± SD (n=4) and analyzed via two-way ANOVA followed by Tukey's post-hoc test for multiple comparisons. Statistically significant differences between equally concentrated $ZnCl_2$ sample groups were considered as $^{\#}p<0.05$, $^{\#\#}p<0.01$, and $^{\#\#\#}p<0.001$ compared with the bLg amyloid treatment group.

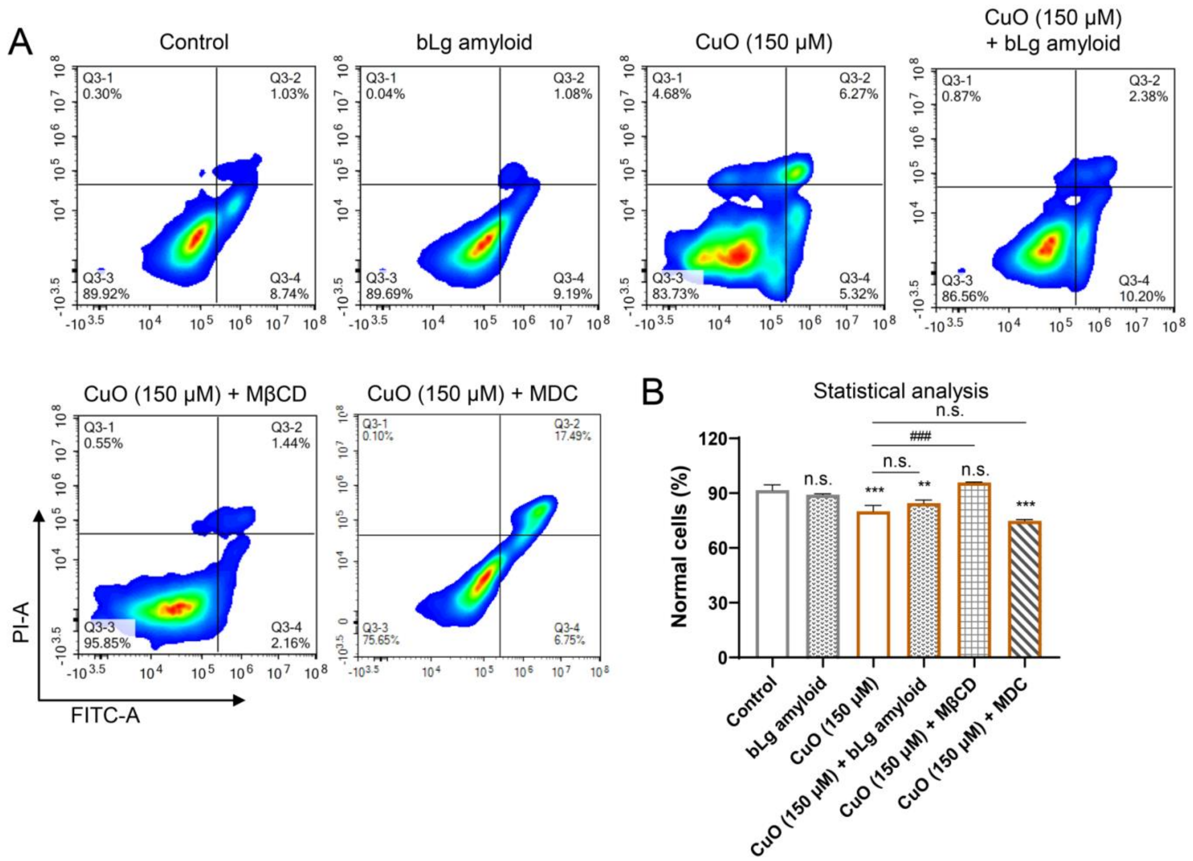

Figure S9. Effects of endocytosis inhibitor pretreatment on the apoptosis and necrosis of HUVECs induced by CuO nanoparticles. (A) Flow cytometry was employed to classify necrotic and apoptotic HUVECs following exposure for 4 h to CuO nanoparticles (150 µM) in the presence and absence of the bLg amyloid (5 mg/mL). Apoptotic cells were identified with Annexin V-FITC labeling, and necrotic cells were labeled with propidium iodide (Q3-1: necrosis. Q3-2: late apoptosis. Q3-3: normal cells. Q3-4: early apoptosis). Cells were treated with inhibitors (MβCD: 5 mM for 2 h; MDC: 10 µM for 1 h) before CuO nanoparticle treatment. (B) Statistical analysis of normal cells (%) in the control and treated cell groups derived from the experimental data presented in panel (A). Data are presented as mean ± SD (n=3) and analyzed via one-way ANOVA followed by Tukey's post-hoc test for multiple comparisons. Significance between the treated and control groups is denoted as *p<0.05, **p<0.01, and ***p<0.001. Accordingly, $^{\#}$p<0.05, $^{\#\#}$p<0.01, and $^{\#\#\#}$p<0.001 represented the significant differences between two treated groups.

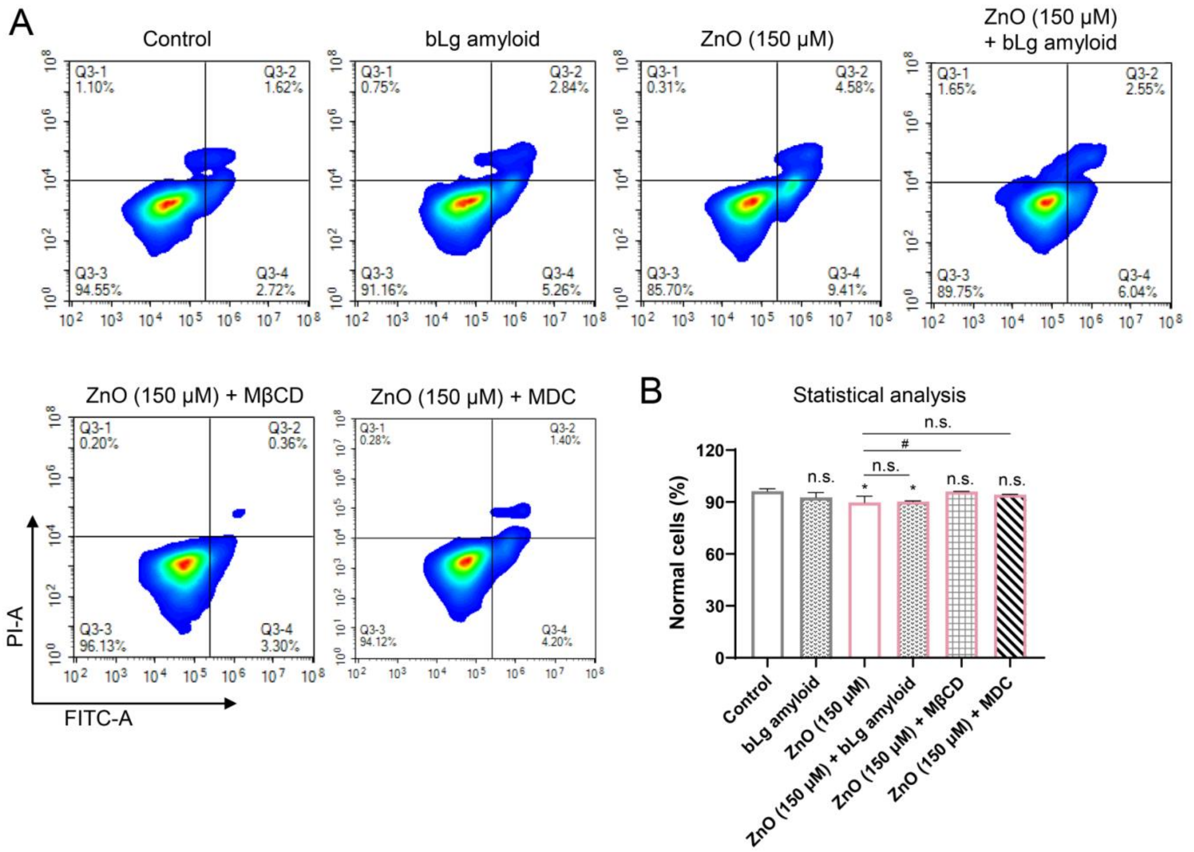

Figure S10. Effects of endocytosis inhibitor pretreatment on the apoptosis and necrosis of HUVECs induced by ZnO nanoparticles. (A) Flow cytometry was employed to classify necrotic and apoptotic HUVECs following exposure for 4 h to ZnO nanoparticles (150 µM) in the presence and absence of the bLg amyloid (5 mg/mL). Apoptotic cells were identified with Annexin V-FITC labeling, and necrotic cells were labeled with propidium iodide (Q3-1: necrosis. Q3-2: late apoptosis. Q3-3: normal cells. Q3-4: early apoptosis). Cells were treated with inhibitors (MβCD: 5 mM for 2 h; MDC: 10 µM for 1 h) before CuO nanoparticle treatment. (B) Statistical analysis of normal cells (%) in the control and treated cell groups derived from the experimental data presented in panel (A). Data are presented as mean ± SD (n=3) and analyzed via one-way ANOVA followed by Tukey's post-hoc test for multiple comparisons. Significance between the treated and control groups is denoted as *$p<0.05$, **$p<0.01$, and ***$p<0.001$. Similarly, $^{\#}p<0.05$, $^{\#\#}p<0.01$, and $^{\#\#\#}p<0.001$ represented the significant differences between two treated groups.

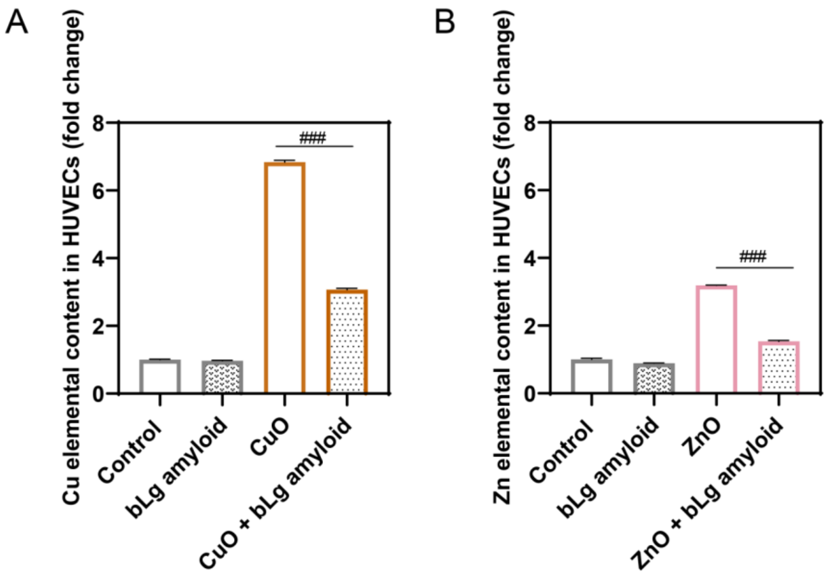

Figure S11. Effect of bLg amyloid on the accumulation of elemental copper (A) or zinc (B) in HUVECs upon exposure to CuO or ZnO nanoparticles. The cells were exposed to CuO or ZnO nanoparticles (150 μM) for 4 h in the presence and absence of the bLg amyloid (5 mg/mL). Statistical analysis through the conduction of t-tests ($^{###}p<0.001$) was used for the evaluation of statistical difference between the two groups. Data are depicted as mean ± SD (n=3).

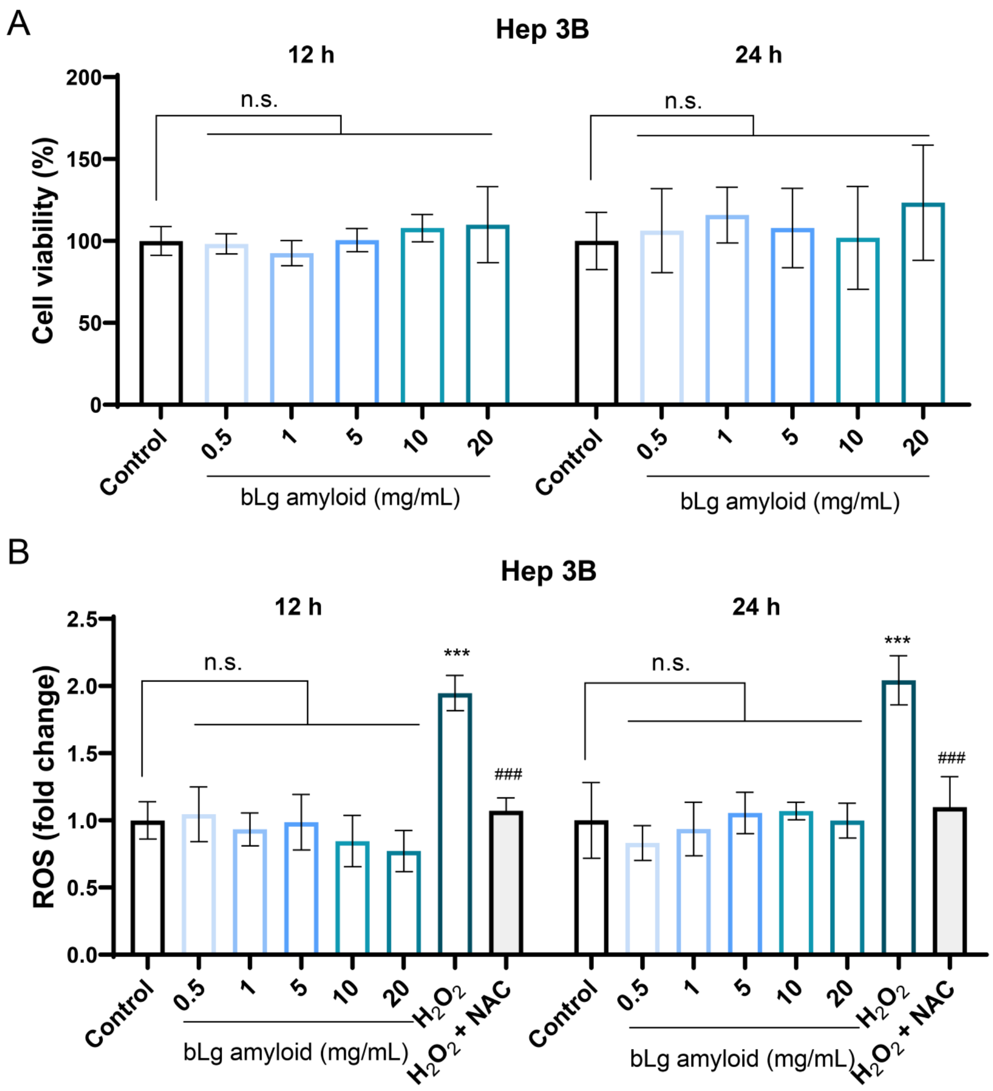

Figure S12. Nonsignificant cell mortality or ROS generation observed in Hep 3B cells upon 12 and 24 h of bLg amyloid treatment. Cell viability (A) and ROS (B) assays following bLg amyloid (0.5, 1, 5, 10, and 20 mg/mL) treatment for 12 and 24 h. The positive control group cells were non- or pre-treated with 5 mM of NAC for 2 h prior to subsequent 2 h H_2O_2 treatment (0.1 mM in culture medium). Data are presented as mean ± SD (n=4) and were analyzed via two-way ANOVA followed by Tukey's post-hoc test for multiple comparisons. Significance between the treated and control groups is denoted by *$p<0.05$, **$p<0.01$, and ***$p<0.001$. $^{###}p<0.001$ represented the significant difference between the H_2O_2 and H_2O_2 + NAC treatment groups.

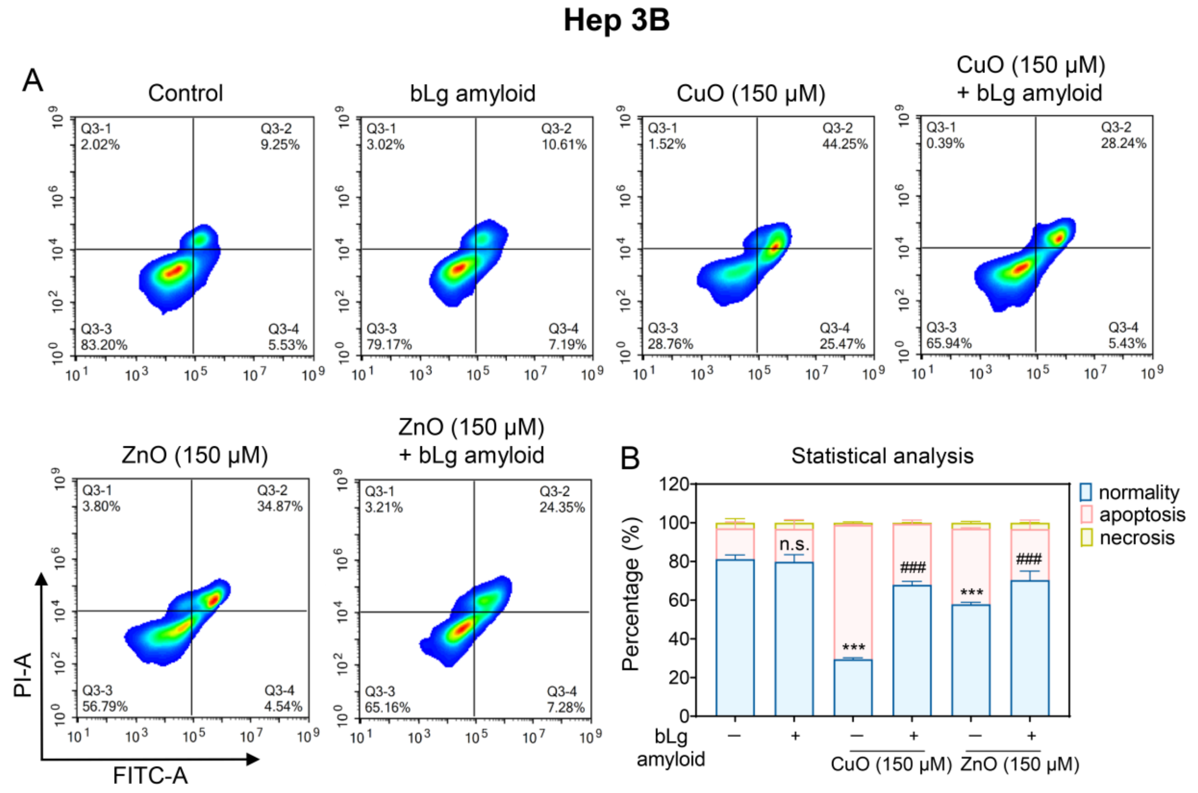

Figure S13. Hep 3B cytotoxicity elicited by CuO and ZnO nanoparticles mitigated by bLg amyloid. (A) Flow cytometry was used to determine necrotic and apoptotic cells upon exposure for 24 h to CuO (150 µM) or ZnO nanoparticles (150 µM) in the presence and absence of the bLg amyloid (5 mg/mL). Apoptotic cells were labeled with Annexin V-FITC, and necrotic cells were labeled with propidium iodide (Q3-1: necrosis. Q3-2: late apoptosis. Q3-3: normal cells. Q3-4: early apoptosis). (B) Statistical evaluation of normal, apoptotic, and necrotic (%) cells in the control and treated cell groups presented in panel (A). Data are presented as mean ± SD (n=3) and were analyzed via two-way ANOVA followed by Tukey's post-hoc test for multiple comparisons. The significance between the treated and control groups is represented as *p<0.05, **p<0.01, and ***p<0.001. #p<0.05, ##p<0.01, and ###p<0.001 denote significant differences between the CuO- or ZnO-nanoparticle-treated groups.

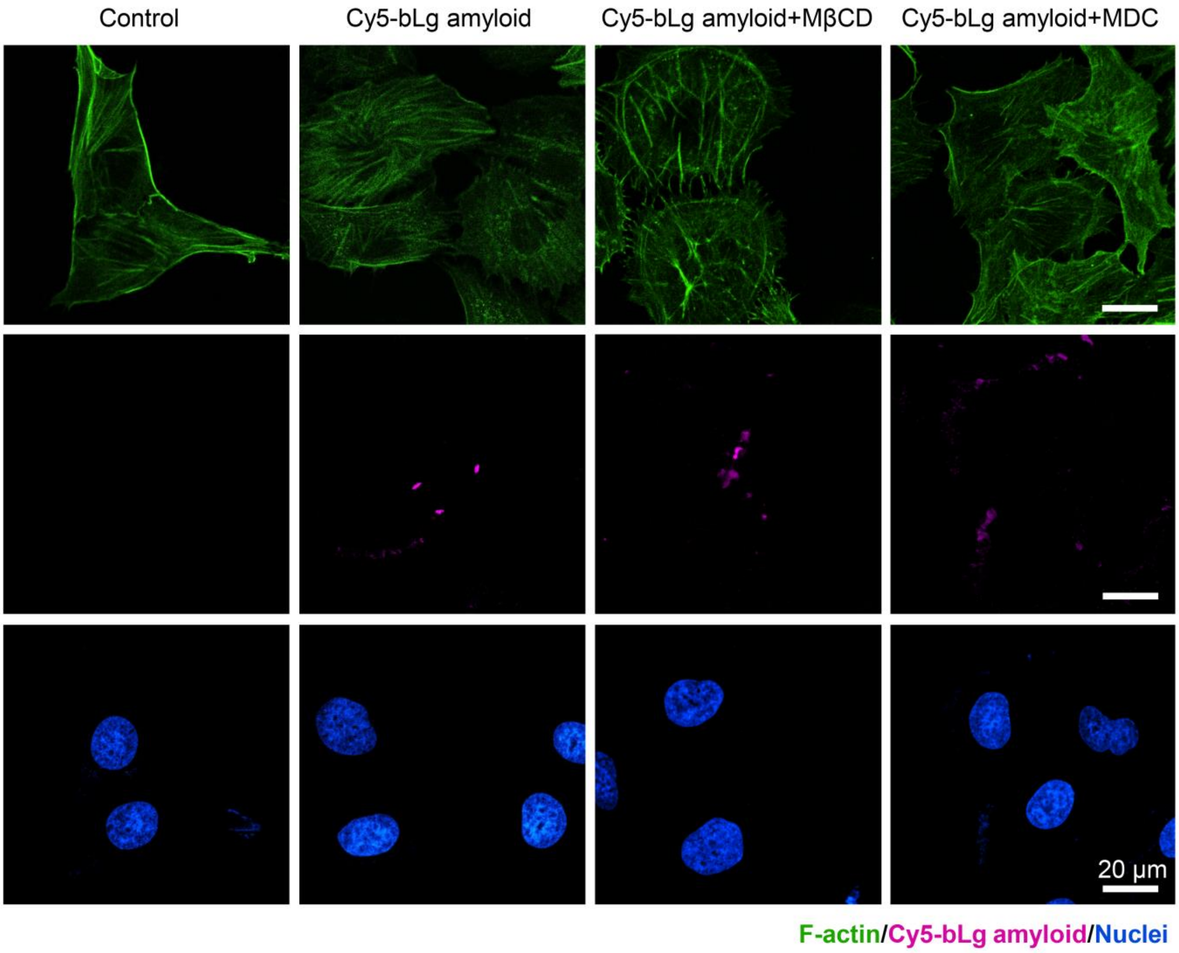

Figure S14. Localization of Cy5-bLg amyloid on the membrane of HUVECs observed using confocal fluorescence microscopy. Single-channel images originated from the merged channel confocal images presented in **Figure 4E**. Sample groups include non-/pre-treated (MβCD/MDC) HUVECs upon exposure for 4 h to Cy5-bLg amyloid (2.5 mg/mL) (n=3). Channels: Nuclei (blue), Cy5-bLg amyloid (purple), F-actin (green). Scale bars: 20 μm.

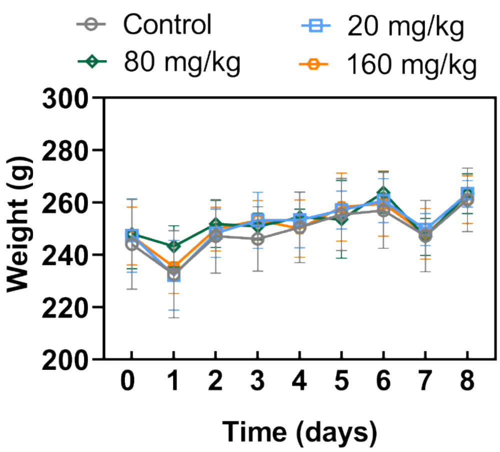

Figure S15. Wistar rats' body weight (g) vs. time. Rats were administered with bLg amyloid at 20, 80, and 160 mg/kg via tail vein injection at day 0, and their body weight was monitored for 8 d. Data are presented as mean ± SD (n=3 per group).

Table S1. Biochemical blood parameters in bLg-amyloid-administered Wistar rats. Blood parameters sampled 7 d after bLg amyloid injection in Wistar rats (n=3 per group). Data are presented as mean ± SD (n=3) and analyzed via one-way ANOVA followed by Tukey's post-hoc test for multiple comparisons. (*p<0.05, **p<0.01, and ***p<0.001).

	bLg amyloid (mg/kg)			
**Parameter (7th day)**	**Control**	**20 mg/kg**	**80 mg/kg**	**160 mg/kg**
ALT (U/L)	62.53±8.09	60.33±2.60	59.63±9.72	57.77±1.56
AST (U/L)	267.70±39.67	239.70±27.00	300.20±59.07	224.33±11.24
BUN (mmol/L)	6.38±0.86	6.10±0.47	7.24±0.73	7.19±0.43
CK (U/L)	2193.10±459.92	1974.03±234.68	2519.63±563.92	1806.57±221.45
GLOB (g/L)	31.20±0.36	30.90±0.22	29.97±1.23	29.37±1.31
AST/ALT	4.30±0.51	3.99±0.58	5.01±0.25	3.89±0.26
WBC (10^9/L)	8.82±0.52	9.39±0.97	8.48±0.78	6.43±0.09 *
RBC (10^12/L)	6.97±0.25	7.06±0.07	6.68±0.04	7.10±0.07
NEUT% (%)	13.27±0.33	14.50±1.61	16.07±2.37	15.80±1.59
LYMPH% (%)	78.17±1.03	77.43±2.02	74.50±2.37	75.57±2.46
MONO% (%)	7.30±1.13	7.03±0.40	8.30±0.24	7.17±0.60
EO% (%)	1.00±0.16	0.87±0.12	1.00±0.29	1.20±0.29
BASO% (%)	0.27±0.05	0.17±0.09	0.13±0.05	0.27±0.05

ALT, alanine aminotransferase; AST, aspartate transaminase; BUN, blood urea nitrogen; CK, creatine kinase; GLOB, globulin; WBC, white blood cell; RBC, red blood cell; NEUT, neutrophils; LYMPH, lymphocytes; MONO, monocytes; EO, eosinophils; BASO, basophils.

References

[1] C. Veerman, H. Ruis, L. M. Sagis, E. van der Linden, *Biomacromolecules* **2002**, *3*, 869.
[2] J.-M. Jung, G. Savin, M. Pouzot, C. Schmitt, R. Mezzenga, *Biomacromolecules* **2008**, *9*, 2477.
[3] M. Jackson, H. H. Mantsch, *Crit. Rev. Biochem. Mol. Biol.* **1995**, *30*, 95.
[4] D. Renuga, J. Jeyasundari, A. S. Shakthi Athithan, Y. Brightson Arul Jacob, *Mater. Res. Express* **2020**, *7*, 045007.
[5] V. Chaurasia, N. Chand, S. K. Bajpai, *J. Macromol. Sci. A.* **2010**, *47*, 309.
